# Bivalent transition metal complexes of triazole pyridine Schiff base with theoretical and biological investigations

**DOI:** 10.1038/s41598-025-15782-3

**Published:** 2025-08-25

**Authors:** Abdullah H. Mannaa, Esam A. Gomaa, Rania R. Zaky, Eslam A. Ghaith, Mahmoud N. Abd El-Hady

**Affiliations:** Chemistry Department, Faculty of Science, Mansoura University, Mansoura, Egypt

**Keywords:** Triazole-pyridine Schiff-base complexes, Spectral characterization, Biological activity, Molecular docking, Chemistry, Materials science

## Abstract

Copper(II), manganese(II), and mercury(II) complexes of 4-amino-5-(2-(1-pyridine-2-yl)ethylidene)hydrazinyl)-4H-1,2,4-triazole-3-thiol (H_2_TAP) were synthesized and characterized using CHN analysis, FT-IR, ^1^H-NMR, ^13^C-NMR, UV–Vis, ESR, MS, PXRD, magnetic moment measurements, molar conductance, and TG/DTA. DFT calculations indicate octahedral geometries and the neutral bidentate or tridentate chelating behavior of the ligand. Cyclic voltammetry revealed the complexes’ redox properties, and Job’s method elucidated stoichiometric compositions in solution. Biochemical assays demonstrated antimicrobial activity against *Escherichia coli*, *Staphylococcus aureus*, and *Candida albicans*. The Mn^II^ complex exhibited potent antitumor activity against HepG-2 cells. Antioxidant and DNA binding studies showed promising results, with docking investigations indicating strong interactions between the ligand/complexes and target proteins (PDB: 1YWN) and DNA (PDB: 8EC1), suggesting therapeutic potential.

## Introduction

Recently, Schiff base complexes incorporating heterocyclic scaffolds particularly triazole and pyridine moieties have attracted significant interest due to their diverse coordination behavior and promising applications in medicinal and organometallic chemistry^1–6^. Hydrazone-based ligands, when functionalized with triazole and pyridine units, serve as tunable polydentate NHC-type ligands with demonstrated biological potential, including antimicrobial, anticancer, antioxidant, and DNA-binding activities^7–14^. These scaffolds also find relevance in catalysis and pharmaceutical synthesis, further highlighting their versatility^15–21^. The molecular hybridization of triazole and pyridine, in particular, offers a unique platform for the development of pincer-like ligands with enhanced metal-binding capacity and multifunctional biological profiles.

Various transition metals in diverse oxidation states play crucial roles in redox enzyme pathways and bio-inorganic processes. Transition metals serve as vital cofactors in various enzymatic processes, promoting electron transfer and stabilizing reaction intermediates. Their capacity to exist in several oxidation states enables participation in intricate biological activities, including those related to respiration and photosynthesis. Furthermore, transition metals enhance the structural integrity of proteins and enzymes, affecting their biological activity and selectivity. Copper is the third most prevalent metal in the human body, behind iron and zinc. Moreover, some enzymes use copper as a cofactor in selective processes; for instance, the active regions of superoxide dismutase include octahedrally coordinated Cu^II^ ions, which facilitate electron transfer to oxygen for ATP energy production^22^. Mn^II^ compounds are notable possibilities for photocatalyst design due to their biocompatibility, environmental compatibility, diverse valence states, and capacity to facilitate numerous electronic excitations^23,24^. Furthermore, Hg^II^ cation compounds are employed in dyes, paper, cosmetics, fluorescent lighting, polymers, and batteries. Moreover, mercuric cations may form complexes that display various structural configurations, characterized by differing coordination numbers^25^.

This study focuses on the synthesis and coordination behavior of a tridentate Schiff base ligand, (Z)-4-amino-5-(2-(1-(pyridin-2-yl)ethylidene)hydrazinyl)-4H-1,2,4-triazole-3-thiol (AHP), with Cu^II^, Mn^II^, and Hg^II^ ions. The ligand is designed to support metal–ligand cooperativity and facilitate electron storage through redox-active coordination sites. We report the synthesis, structural characterization, and biological evaluation of the resulting complexes, along with molecular docking studies to rationalize their bioactivity at the molecular level.

## Material and methods

### Chemicals

The chemicals CuCl_2_.2H_2_O, MnCl_2_.4H_2_O, Hg(OAc)_2_, DMSO, KCl, 4-amino-5-hydrazinyl-4*H*-1,2,4-triazole-3-thiol, 1-(pyridin-2-yl)ethan-1-one, sulfuric acid, absolute MeOH, and EtOH were utilized out of any further processing and supplied by Sigma-Aldrich, and BDH.

### Synthesis of H_2_TAP ligand and complexes

**Scheme 1:**
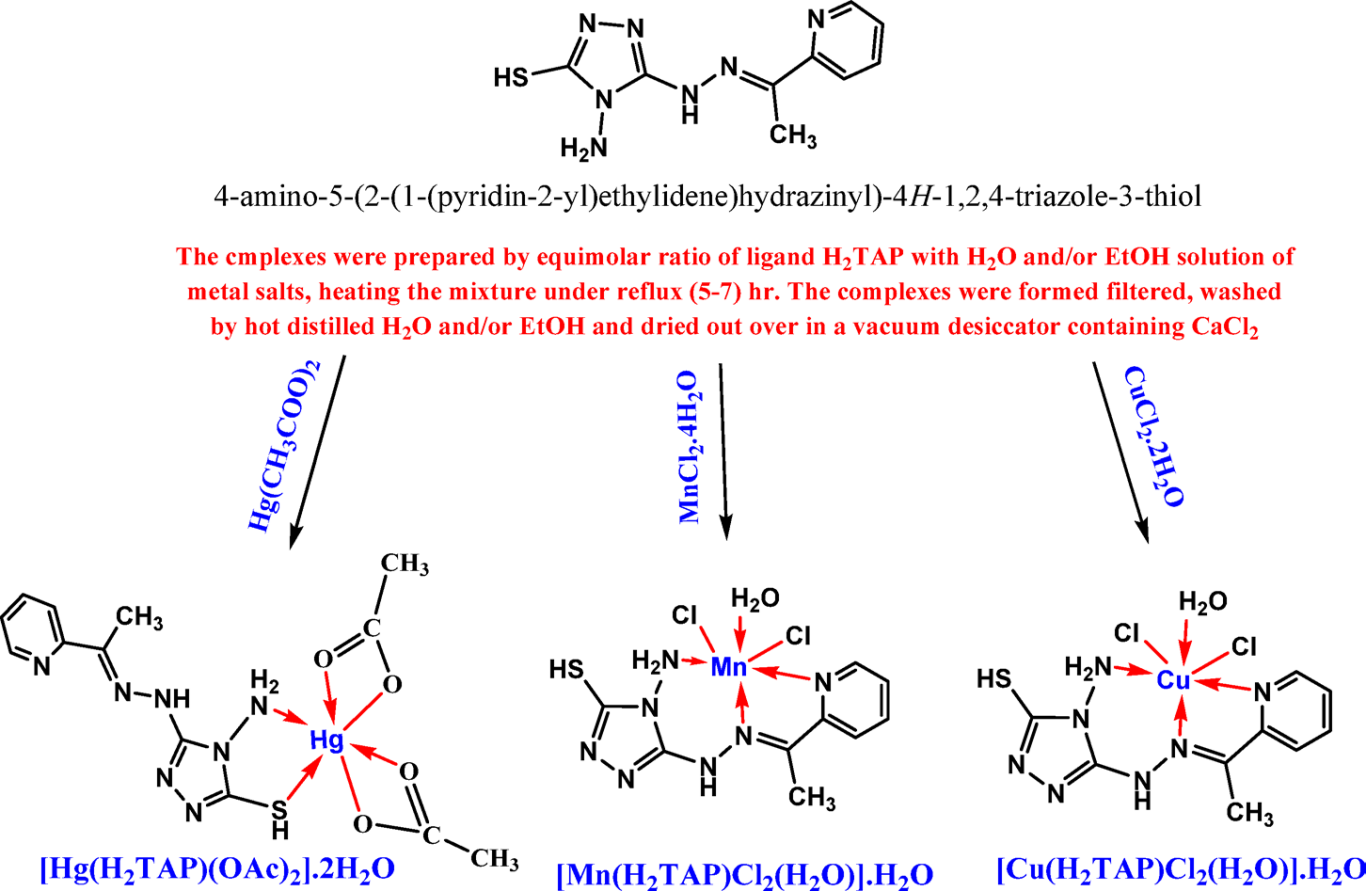
The outline synthesis of H_2_TAP ligand and its metal complexes.

The synthesis of 4-amino-5-(2-(1-(pyridin-2-yl)ethylidene)hydrazinyl)-4H-1,2,4-triazole-3-thiol ligand (H₂TAP) was accomplished by ultrasonic heating of 4-amino-5-hydrazinyl-4H-1,2,4-triazole-3-thiol (1 mmol; 0.146 g) with 1-(pyridin-2-yl)ethan-1-one (1 mmol; 0.121 g) in Methanol (20 mL) at 80 °C for 45 min. The resulting Orange precipitate was collected by filtration, washed with hot CH_3_OH, and dried under vacuum to afford the ligand in 78% yield^26,27^. Then, CuCl_2_.2H_2_O (1mmol; 0.170 g), MnCl_2_.4H_2_O (1mmol; 0.198 g) and Hg(OAc)_2_ (1mmol; 0.319 g) salts were used with the ligand in equimolar ratios producing the metal chelates according to Scheme 1.

### Instruments

The elemental compositions (C, N, H, and S) of the H_2_TAP ligand and metal complexes were analyzed at the regional Center for Mycology and Biotechnology (RCMB), Al Azhar University, Egypt. Also, the metal cations’ contents were estimated by complexometric titrations. Whereas, the compounds’ structures were elucidated via the following instruments: FT-IR, ^1^H/^13^C-NMR, UV–Visible, MS, EPR, PXRD, TG/DTA, and magnetic susceptibility balance (Table S1).

### Cyclic voltammetry technique

In this study, CuCl_2_.2H_2_O, MnCl_2_.4H_2_O, Hg(OAc)_2_, DMSO and KCl were used without any treatment. In a cell containing 0.1M KCl solution, the voltage was controlled using a DY2100 potentiostat that supported three electrodes—a glassy carbon working electrode (GCE), a platinum auxiliary electrode, and an Ag/AgCl reference electrode. To study investigate the electrochemical behavior of the H_2_TAP ligand and Cu^II^, Mn^II^ and Hg^II^ complexes has been conducted using 0.1M of KCl solution as the supporting electrolyte dissolved in 50% (DMSO-water) mixed solvent^28,29^. All cyclic voltammograms were recorded according to the IUPAC convention at scan rate 0.05 V/s and temperature 293.15K.

### Job’s method

This study used a modified version of Job’s continuous variation approach to examine the interaction between metal ions and the ligand. A series of solutions was formed by combining equimolar solutions of metal ions and the ligand in varying proportions, maintaining a constant total molar concentration of 10^–3^ M, conducted at room temperature (25 °C). A graph depicting the absorbance of the solutions at the specified wavelength against the mole fraction of the metal ions shows a peak at the anticipated molar ratio of the most stable complexes^30,31^.

### Gaussian studies

The Gaussian 09W software was adjusted to investigate the three-dimensional-optimized molecular structures of the compounds using density functional theory (DFT) with correlation functional (B3LYP) with a combination of 6–311 + G (d, p)/LANL2DZ for the complexes and 6–311 + G (d, p) as the basis set for the ligand^32–34^.

### Antimicrobial activity

The antibacterial and antifungal activities of the synthesized compounds were evaluated using the disc diffusion method^35,36^, as illustrated in Scheme S1. The microbial strains tested included *Staphylococcus aureus* (Gram-positive), *Escherichia coli* (Gram-negative), and *Candida albicans* (fungal). Sterile filter paper discs (6 mm) were impregnated with 20 µL of each compound solution at a concentration of 100 µg/mL in DMSO and placed on the surface of Muller–Hinton agar (for bacteria) and Sabouraud dextrose agar (for fungi) pre-inoculated with the microbial suspensions (10⁶ CFU/mL). The plates were incubated at 37 °C for 24 h (bacteria) and 28 °C for 48 h (fungi). *Ciprofloxacin* (5 µg/disc) and *clotrimazole* (10 µg/disc) served as positive controls, while DMSO was used as a negative control. The inhibition zones were measured in millimeters, and the antimicrobial activity was expressed as a percentage relative to the standard drugs. In addition, the minimum inhibitory concentration (MIC) was determined using the broth microdilution method with two-fold serial dilutions of the antibiotic (64 to 0.5 µg/mL) in Mueller–Hinton broth. Bacterial suspensions were standardized to 5 × 10^5^ CFU/mL and incubated at 37 °C for 18–20 h before visual assessment of growth inhibition.

### Antioxidant activity (ABTS Assay)

The antioxidant activity was assessed using the ABTS radical cation decolorization assay, with L-ascorbic acid serving as the reference antioxidant. ABTS·⁺ radicals were generated by reacting 7 mM ABTS solution with 2.45 mM potassium persulfate and allowing the mixture to stand in the dark at room temperature for 12–16 h. The working solution was diluted to an absorbance of 0.700 ± 0.020 at 734 nm. Test compounds were prepared in methanol at concentrations of 100, 80, 60, 40, 20, and 10 µM. Each sample (100 µL) was mixed with 1 mL of ABTS^+^ solution, incubated for 30 min at room temperature in the dark, and absorbance was measured at 734 nm (Scheme S2). All experiments were performed in triplicate. The percentage inhibition was calculated, and IC₅₀ values were derived from the inhibition curves. *L-ascorbic acid* was used as the positive control, and methanol served as the blank^37,38^.

### DNA-binding assay

DNA-binding affinity was evaluated using the methyl green (MG) displacement method^39,40^ as shown in Scheme S3. A fixed concentration of calf thymus DNA (CT-DNA, 50 µM) and MG (20 µM) were incubated together in Tris–HCl buffer (10 mM, pH 7.2) to form a stable DNA-MG complex. Subsequently, increasing concentrations of test compounds (10–50 µM) were added, and the mixtures were incubated at room temperature for 30 min. The displacement of MG was monitored spectrophotometrically by recording the decrease in absorbance at 630 nm. A reduction in absorbance indicates effective displacement of MG from DNA, implying the binding affinity of the compound to the DNA duplex. Control samples without compounds were used to establish the baseline absorbance of the DNA-MG complex.

#### MTT assay (anticancer activity)

The cytotoxic effects of the synthesized compounds were determined against human liver carcinoma cells (HepG2) using the MTT assay protocol^41,42^. Cells were seeded in 96-well plates at a density of 5 × 10^3^ cells/well and incubated in DMEM medium supplemented with 10% FBS and 1% penicillin–streptomycin at 37 °C in a humidified atmosphere with 5% CO₂. After 24 h attachment, cells were treated with various concentrations (100, 50, 25, 12.5, 6.25 µM) of the test compounds dissolved in DMSO (final DMSO concentration < 1%) and incubated for an additional 48 h. Following treatment, 20 µL of MTT solution (5 mg/mL in PBS) was added to each well and incubated for 4 h. The medium was removed, and 150 µL of DMSO was added to dissolve the resulting formazan crystals. Absorbance was recorded at 570 nm using a Gen5 microplate reader (BioTek). Untreated cells were used as the negative control, while cells treated with *Cis-platin, Doxorubicin, and Sorafenib* (10 µM) served as the positive control, as illustrated in Scheme S4.

#### Molecular docking

Molecular docking simulations of compounds against protein receptors 1YWN and 8EC1, which are overexpressed in human hepatocellular carcinoma and DNA, were performed via MOE software. All proteins were downloaded through the protein data bank as PDB files. H_2_O molecules associated with proteins were eliminated to prevent interference with the investigation of docking^43,44^.

## Result with discussions

### Structural characterization

#### Physicochemical properties

The results of the elemental analysis and physical characteristics are presented in Table 1, where the calculated and observed percentages corroborate the proposed chemical formulae: CuC_9_H_15_Cl_2_N_7_O_2_S, MnC_9_H_15_Cl_2_N_7_O_2_S, and HgC_13_H_21_N_7_O_6_S. A single cation of copper, manganese, and mercury with a stoichiometry of (1 metal: 1 ligand) was incorporated into the complex. This made five- or six-membered ring chelates (Scheme 1). At room temperature in DMSO, the molar conductance values of all the complex solutions (10^−3^ M) ranged from 22.6 to 49.9 μS cm^2^ mol^−1^. The results show that Cl^−^ and OAc^−^ are coordinated to the metal ions in the Cu^II^, Mn^II^, and Hg^II^ complexes. This means that they are non-electrolytic, as shown in Table 1.

**Table 1:** Physical properties and elemental analyses of H_2_TAP ligand and its complexes.

Compound	M.wt	Physical properties			Elemental analysis found (calculated)					
		color	m.p.(oc)	Molar conductance (Μs cm^2^ mol^−1^)	C%	H%	N%	S%	M%	Cl-%
H2TAP	249.30	orange	248–250	–	43.18 (43.36)	4.53 (4.45)	39.61 (39.33)	12.79 (12.86)	–	–
[Cu(H2TAP)(Cl2)(H2O)]·H2O	419.77	Dark green	> 300	22.6	26.50 (25.75)	3.89 (3.60)	23.31 (23.36)	(7.64)	15.62 (15.14)	16.11 (16.89)
[Mn(H2TAP)(Cl2)(H2O)]·H2O	411.16	Yellow orange	> 300	49.8	25.68 (26.29)	3.84 (3.68)	23.47 (23.85)	7.85 (7.80)	13.76 (13.36)	17.48 (17.24)
[Hg(H2TAP)(OAc)2]·2H2O	604.00	yellow	> 300	24.9	26.57 (26.82)	3.26 (3.25)	24.38 (24.33)	7.73 (7.95)	24.53 (24.88)	–

#### FT-IR

The FT-IR analysis of the H_2_TAP ligand, detailed in Table S2 and Fig. S1, identifies several significant absorption bands that elucidate the ligand’s structural features and functional groups. The observed bands at stretching frequencies 1607 cm^−1^, 1641 cm^−1^, 3302 cm^−1^, 2890 cm^−1^, and 859 cm^−1^ correspond to specific functional groups within the ligand: azomethine ν(C=N) _Schiff-Base_, ν(C=N) _triazole_, amine ν(NH_2_), thiol ν(SH), and the ring breathing mode of pyridine, respectively (Fig. S1a).

The spectra of Cu^II^ and Mn^II^ complexes indicate that (H_2_TAP) functions as a neutral tridentate ligand via v(C=N)_Schiff-Base_, ν(NH_2_), and pyridine nitrogen, as discussed in various reviews: (i) the shift of azomethine group to lower wavenumber 1595 and 1600 cm^−1^; (ii) the displacement of amine group to lower wavenumber 3242 and 3246 cm^−1^; (iii) the presence of pyridine ring vibrations at 866 and 867 cm^−1^; (iv) the emergence of new ν(Cu–N) at 498 cm^−1^ and ν(Mn–N) at 481 cm^−1^ (Fig. S1b,c). The analysis of the Hg^II^ complex reveals the presence of neutral bidentate chelation involving both thiol (–SH) and amine (–NH_2_) groups, as evidenced by: (i) the observed shift of the thiol and amine groups to lower wavenumbers at 2859 cm^−1^ and 3287 cm^−1^, respectively; (ii) the emergence of new bands at 457 cm^−1^ for ν(Hg–N), 477 cm^−1^ for ν(Hg–S), 1530 cm^−1^ for (COO^−^)_sym_, and 1453 cm^−1^ for (COO^−^)_assym_ (Fig. S1d).

#### Bond lengths correlation

**Fig. 1:**
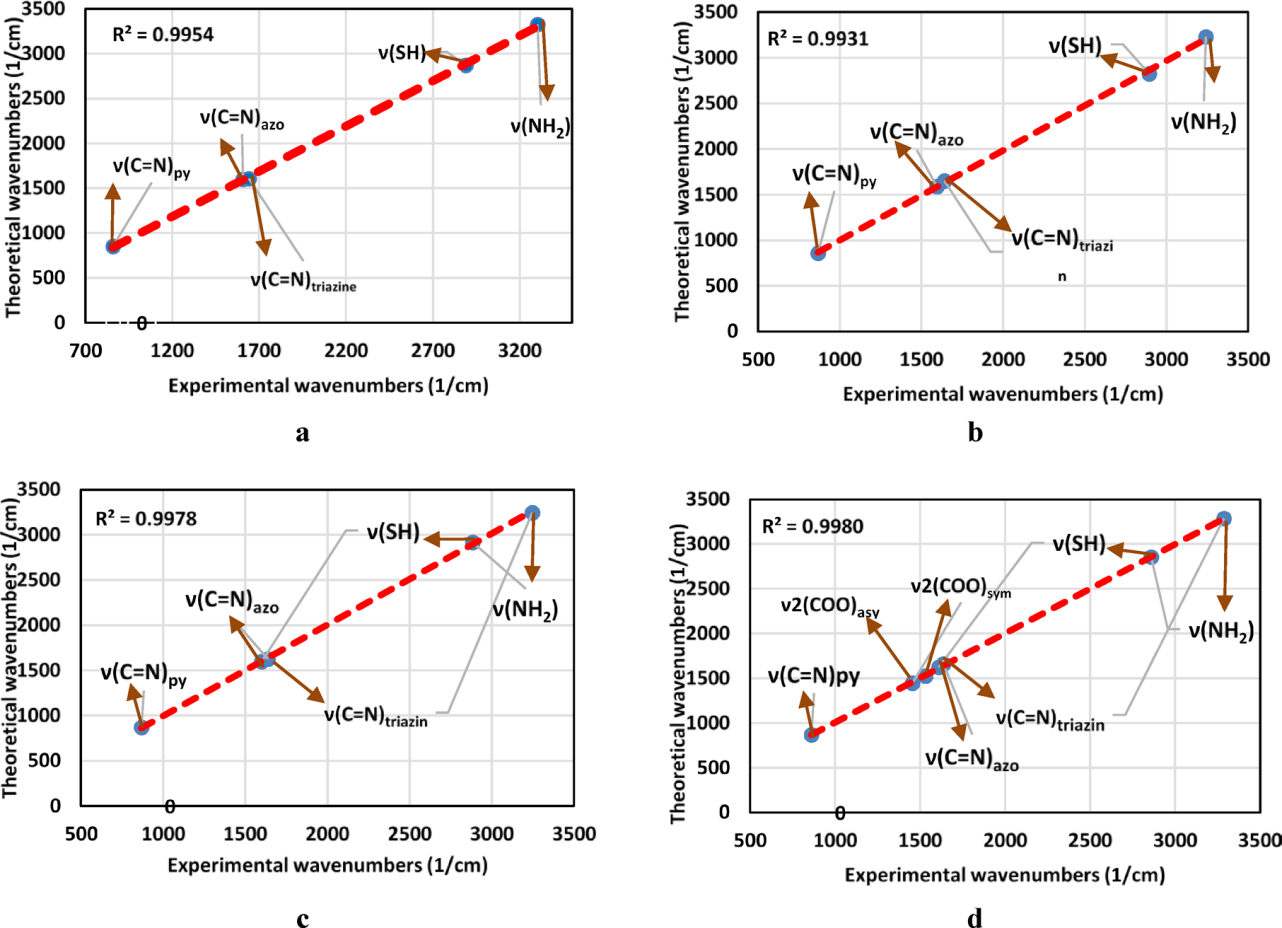
The Experimental and theoretical IR spectrum of )**a**) H_2_TAP ligand, (**b**) [Cu(H_2_TAP)( H_2_O)(Cl_2_)]·H_2_O, (**c**) [Mn(H_2_TAP)(H_2_O)(Cl_2_)]·H_2_O and (**d**) [Hg(H_2_TAP)(OAc)_2_]·2H_2_O.

An infrared spectroscopic correlation analysis of experimental and theoretical absorption bands was carried out. Theoretical absorption bands were calculated using DFT frequency data, while experimental absorption bands were identified using observed IR spectra of solid compounds. Statistical relationships were examined using convergence diagrams and scatter plots^45^. Figures 1 and S2 and Table 2 show a high positive linear association across all samples, with R^2^ values ranging from 0.9931 to 0.9980.

**Table 2:** The correlation between experimental (theoretical) IR bands.

Compound	R^2^	ν(C=N)_azo_	ν(C=N)_triazine_	ν(NH_2_)	ν(SH)	ν(C=N)_py_	ν(COO^−^)_sym_	ν(COO^−^)_asy_
H_2_TAP	0.9954	1607 (1600)	1641 (1605)	3302 (3325)	2890 (2869)	859 (853)	–	–
[Cu(H_2_TAP)(Cl_2_)(H_2_O)]·H_2_O	0.9931	1595 (1589)	1640 (1649)	3242 (3230)	2894 (2823)	866 (857)	–	–
[Mn(H_2_TAP)(Cl_2_)(H_2_O)]·H_2_O	0.9978	1600 (1596)	1637 (1626)	3246 (3245)	2883 (2918)	867 (871)	–	–
[Hg(H_2_TAP)(OAc)_2_]·2H_2_O	0.9980	1610 (1623)	1636 (1660)	3287 (3286)	2859 (2854)	860 (868)	1530 (1525)	1453 (1445)

#### MS and NMR

The molar mass of the H_2_TAP ligand was represented by a molecular ion peak of [M]^+^ (m/z = 249.74), as shown in Fig. S3. The molecular weight of 249.30 agrees with the proposed formula (C_9_H_11_N_7_S). The ^1^H-NMR spectrum of H_2_TAP as illustrated in Fig. S4a showed the following observations: (i) amine protons (N**H**_**2**_) at chemical shift (δ) 4.037 ppm, (ii) proton of (N**H**) group existed at (δ) 9.909 ppm, (iii) thiol proton (S**H**) manifested at (δ) 14.369 ppm; (iv) methyl protons (C**H**_**3**_) arose in the range of (δ) 2.520–2.383 ppm; (v) aromatic protons occurred within the range of (δ) 7.55–8.73 ppm. The ^13^C-NMR spectrum of H_2_TAP as shown in Fig. S5a demonstrated the following: (i) methyl carbon as a sharp singlet at (δ) 12.39 ppm; (ii) aromatic carbons are observed at (δ) 149.55, 146.15, 128.28, 125.27, 122.61 ppm; (iii) sharp peaks at (δ) 138.27, 149.75, and 165.12 ppm are attributed to azomethine carbon (**C**=N), triazole carbon (**C**=N), and (S–**C**=N); respectively.

The ^1^H-NMR spectrum of [Hg(H_2_TAP)(OAc)_2_]0.2H_2_O as illustrated in Fig. S4b showed the following observations: (i) amine protons (N**H**_2_) at chemical shift (*δ*) 4.022 ppm (ii) (N**H**) proton at (*δ*) 10.343 ppm; (iii) thiol proton (S**H**) at chemical shift (*δ*) 14.998 ppm; (iv) methyl protons (C**H**_3_) at (*δ*) 2.399–2.490 range ppm, (v) aromatic protons appeared in the (*δ*) 7.425–8.611 ppm region. The ^13^C-NMR spectrum of [Hg(H_2_TAP)(OAc)_2_]0.2H_2_O as clarified in Fig. S5b detected the following criteria: (i) methyl carbon as sharp singlet at (*δ*) 12.37 ppm; (ii) aromatic carbons in (*δ*) 150.82, 148.42, 137.15, 124.37, 120.79 ppm region; (iii) sharp peaks at (*δ*) 138.76, 153.99, and 147.43 ppm are assigned to azomethine carbon (**C**=N), triazole carbon (**C**=N), and (S–**C**=N); respectively, as shown in Table S3.

#### UV–Visible and magnetic moment

The UV–Visible spectrum of [Cu(H_2_TAP)(H_2_O)(Cl_2_)].H_2_O displayed a broad bands at 16,129 and 14,184 cm^−1^ corresponding to ^2^B_1_g → ^2^Eg and ^2^B_1_g → ^2^A_1_g transitions and the magnetic moment (*μeff* = 1.91 B.M.) identifying an octahedral arrangement configuration in d^9^ configuration^46^. The spectra of [Mn(H_2_TAP)(H_2_O)(Cl_2_)]·H_2_O exhibited two bands at 27,777 and 19,084 cm^−1^, attributed to the ^4^T_1_g → ^4^A_2_g(F) and ^4^T_1_g → ^3^T_1_g(P) transitions, respectively^47^. The magnetic moment value indicates a high spin octahedral configuration (μeff = 5.82 B.M.), as illustrated in Table S4 and Fig. S6.

#### EPR examination

**Fig. 2:**
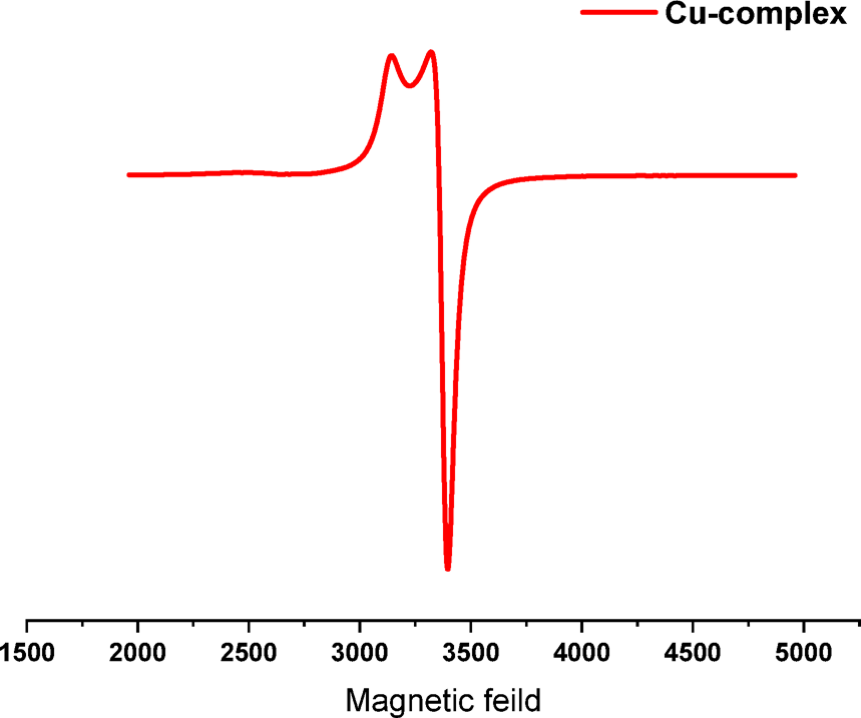
ESR spectra of [Cu(H_2_TAP)(H_2_O)(Cl_2_)]·H_2_O complex.

The electron paramagnetic resonance (EPR) spectra of the Cu^II^ complex (Fig. 2 and Table S5) was investigated using a Bruker EMX spectrometer operating at room temperature at a frequency of 9.685 GHz in the X-band, with a modulation frequency of 100 kHz. The Cu^II^ complex, characterized by (S = ^**1**^**/**_**2**_, I = ^**3**^**/**_**2**_) Spin Hamiltonian parameters, exhibited a four-line hyperfine pattern alongside axial symmetry in the calculated g-tensor parameters (g_||_= 2.142 > g_⊥_ = 2.064 > g_e_ = 2.0023), determined using Eq. (1) and the assessed G-factor via Eq. (2) ^48^. This pattern implies an octahedral geometry within the d_x2-y2_ ground state^49^. The h, v, B, and µ represent the Planck constant (6.626 × 10^–34^ J), frequency (9.865 MHz), Bohr magneton (9.27 × 10^–24^ J/mT), and applied magnetic field (mT), respectively. In a solid-state complex, G denotes the exchange interaction among Cu^II^ centers. If G exceeds 4, the exchange contact is minimal; conversely, if G is less than 4, significant interaction occurs in the Cu^II^ complex^50^. Furthermore, the parameters of the parallel and perpendicular hyperfine components (A_||_= 0.010 and A_⊥_ = 0.093) were ascertained using spectral analysis. F-factors are utilized to quantify the extent of distortion in Cu^II^ complexes. If the number is between 105 and 135, the complex will adopt a square-planar geometry; however, values over 135 will result in an octahedral configuration. The Cu^II^ sample yields (F = 205.96), indicating octahedral geometry.

1 $${\text{g }} = {\text{ h}}\upnu /\upmu {\text{B}}$$

2 $${\text{G }} = \, \left( {{\text{g}}_{||} {-}{ 2}} \right)/({\text{g}}_{ \bot } {-}{ 2}) \, = { 4}$$

3 $${\text{F }} = {\text{g}}_{||} /{\text{A}}_{||}$$

To determine the coefficients of molecular orbitals, the covalent in-plane σ-bonding (α^2^) and covalent in-plane π-bonding (β^2^) were determined using Eqs. (4 and 5). (E) Denotes the electronic transition energy. The free Cu^II^ has a value of λ = − 828 cm^−1^. The factor (α^2^ = 1) indicates complete ionic character, while (α^2^ = 0.5) signifies total covalent bonding, and lower values are negligible. The in-plane π-bonding covalence intensifies when the β^2^ component diminishes^51^. The Cu^II^ complex demonstrates robust in-plane π-bonding, as evidenced by α^2^ = 0.495 and β^2^ = 0.937. This effect is anticipated as the ligand possesses orbitals that interact with the copper’s d_xy_ orbitals.

4 $$\alpha^{{2}} = \, ({\text{A}}_{||} /0.0{36}) \, + \, ({\text{g}}_{||} {-}{ 2}.00{23}) \, + { 3}/{7}({\text{g}}_{ \bot } {-}{ 2}.00{23}) \, + \, 0.0{4}$$

5 $$\beta^{2} = (g_{||} {-} \, 2.0023) \, E/ \, {-} \, 8\lambda \alpha^{2}$$

#### TGA/DTA studies

Thermal assessment TG/DTA was conducted from ambient temperature to 800 °C in a nitrogen atmosphere to ascertain whether the water molecules were coordinated or crystallized, as illustrated in Table S6 and Fig. S7 within Scheme S5, which delineates the stages of thermal breakdown.

TG curve of the [Cu(H_2_TAP)(H_2_O)(Cl_2_)].H_2_O complex (Fig. S7a) exhibited a weight loss of 8.58% within the temperature range of 27 to 195 °C, attributed to the elimination of two water molecules involved in crystallization and coordination. The curve indicated a weight loss of 16.93% in the temperature range of 195 to 255 °C, corresponding to the release of Cl_2_ gas. The subsequent analysis identified a weight loss of 27.42% within the temperature range of 255 to 328 °C, corresponding to the (C_2_H_3_N_4_S) fragment. During the temperature range of 328 to 799 °C, the TG curve indicated a decomposition rate of 24.32%, which is linked to the complete breakdown of the more firmly anchored organic molecule (C_7_H_4_N) and the rupture of the chelate bond. This process resulted in the formation of the Cu(NH_2_)_2_ molecule, constituting 22.75% of the complex’s initial mass (Scheme S5a).

The TG curve of [Mn(H_2_TAP)(H_2_O)(Cl_2_)]·H_2_O chelate (Fig. S7b) manifested a weight loss of 4.22% across the temperature range of 27 to 175 °C, which is associated with one water molecule of crystallization. In the forthcoming stage, occurring at temperatures between 175 and 338 °C, there was a loss of 21.99% of two coordinating water molecules and Cl_2_ gas. In the temperature range of (338–550) °C, a mass loss of 28.11% occurred due to the removal of the (C_2_H_3_N_4_S) fragment. Throughout the temperature range of 550 to 799 °C, the TG curve replied a decomposition rate of 24.30%, which was linked to the total breakdown of the more firmly anchored organic molecule (C_7_H_4_N). The mass percentage of the remaining component of one molecule of Mn(NH_2_)_2_ was found to be 21.38% (Scheme S5b).

#### PXRD analysis

**Fig. 3:**
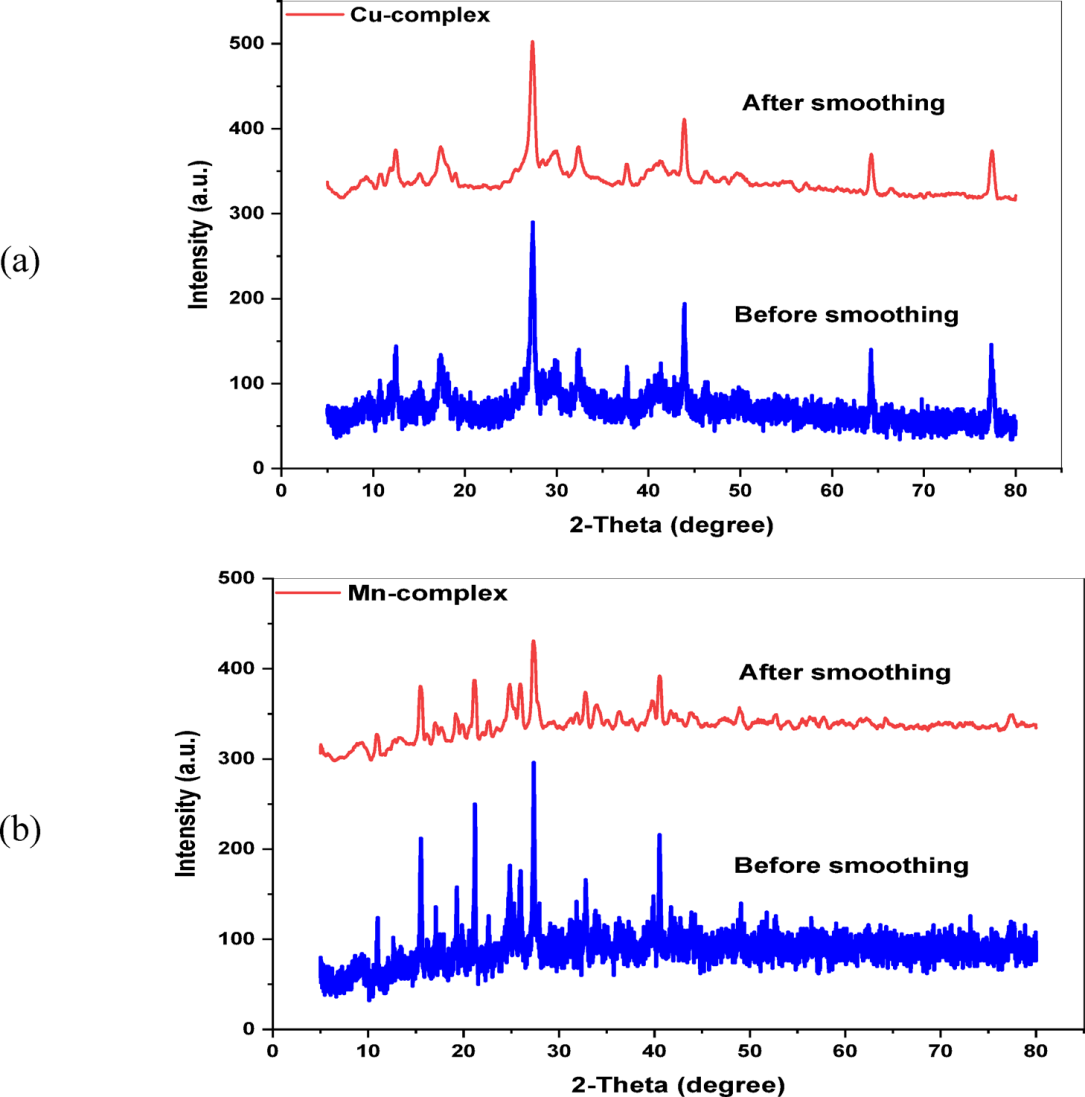
PXRD patterns of (**a**) [Cu(H_2_TAP)(H_2_O)(Cl_2_)]·H_2_O and (**b**) [Mn(H_2_TAP)(H_2_O)(Cl_2_)]·H_2_O.

X-ray powder diffraction examinations were performed at room temperature using the Rigaku Ultima IV, which utilizes Cu anode radiation (Kα, λ = 1.5418 Å) across a 2θ range of 4° to 70° (Fig. 3). A diffraction pattern of the crystal phase was acquired for the isolated complexes. The Debye–Scherrer equation, S = 0.9λ/(β cos θ), can be employed to determine the size of a crystalline sample (S) at the most intense peak, where β represents the FWHM, λ = 1.5406 Å, and θ denotes the diffraction angle. Given n equals one, the Bragg equation nλ = 2 d sin(θ) can be employed to determine the values of d-spacing^52^. The results in Table 3 indicate that Cu^II^ and Mn^II^ complexes exhibit crystalline phase systems with crystal sizes of 19.311 nm and 36.387 nm, respectively (Fig. 3).

**Table 3:** XRD data of Cu^II^ and Mn^II^ metal complexes.

Compound	2θ	d–Spacing (Å)	FWHM (*β*)	Crystal size (nm)
[Cu(H_2_TAP)(Cl_2_)(H_2_O)]·H_2_O	27.327	3.260	0.423	19.311
[Mn(H_2_TAP)(Cl_2_)(H_2_O)]·H_2_O	27.327	3.260	0.225	36.387

**Fig. 4:**
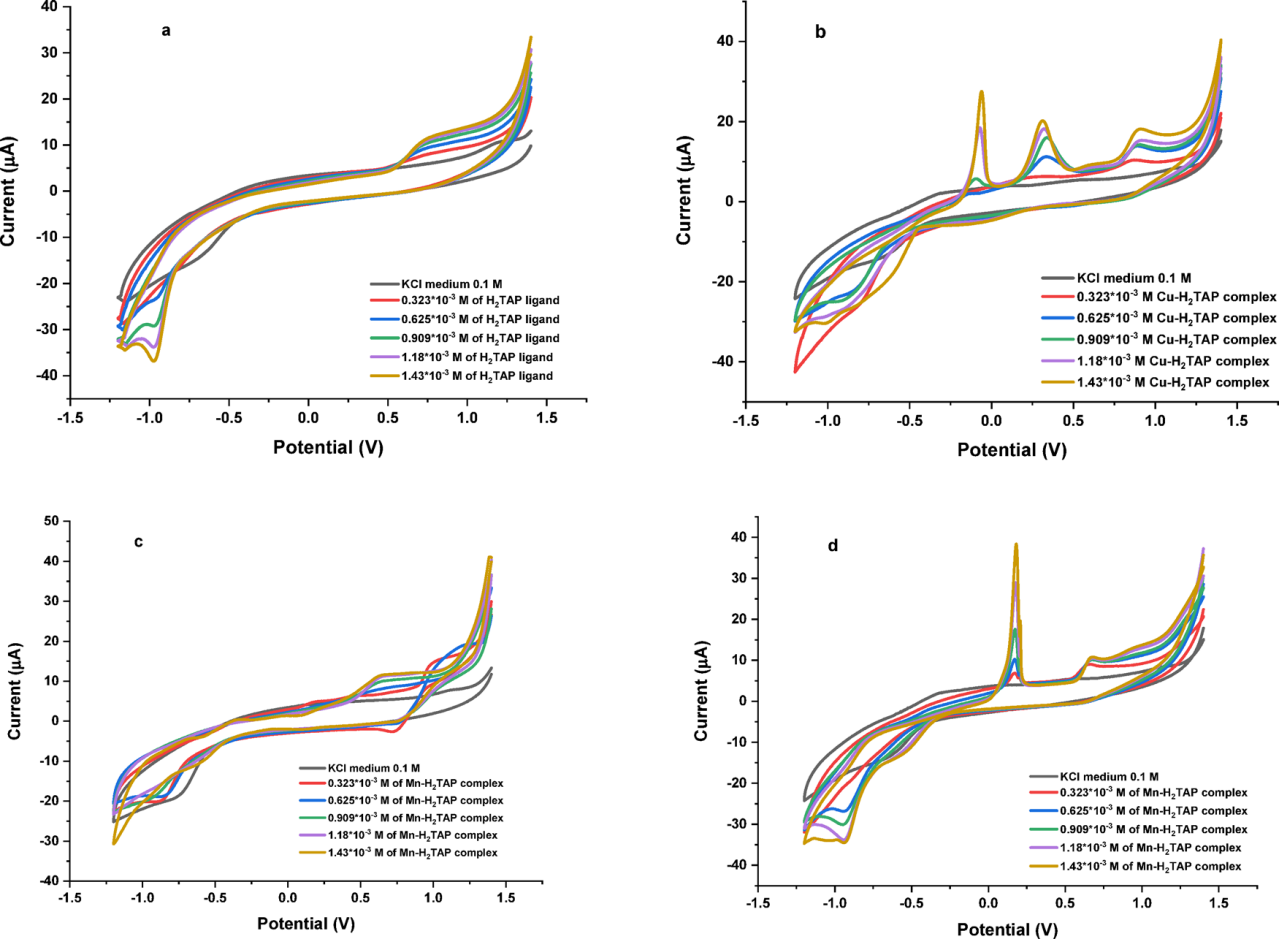
Cyclic voltammogram of )**a**) H_2_TAP ligand, (**b**) [Cu(H_2_TAP)(H_2_O)(Cl_2_)]·H_2_O, (**c**) [Mn(H_2_TAP)(H_2_O)(Cl_2_)]·H_2_O and (**d**) [Hg(H_2_TAP)(OAc)_2_].2H_2_O complexes.

#### Job’s method (continuous variations)

**Fig. 5:**
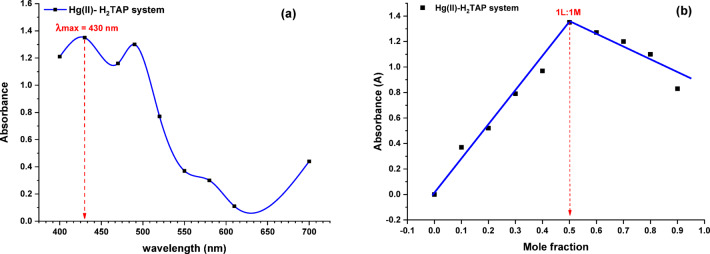
(**a**) Maximum wavelength (λmax) and (**b**) stoichiometric determination of Hg^II^ complex using Job’s continuous variation method of Hg^II^ and H_2_TAP ligand solution.

Job’s method of continuous variation was utilized to ascertain the stoichiometry and the thermodynamic stability constant of the Hg^II^ complex. Stock solutions of concentrations (1 × 10^−3^ mol/L) for both Hg^II^ ions and the H₂TAP ligand were prepared and introduced to prepare mole fractions (Table 4). Then the measured absorbance for each sample was plotted against the corresponding mole fraction to determine the composition that resulted in the highest absorbance, signifying maximum complex formation. The absorbance of all measured solutions was recorded at λmax = 430 nm—was chosen based on the absorbance–wavelength relationship depicted in Fig. 5a, and Table 5a—to ensure precise and consistent detection. In Fig. 5b and Table 5b, the plot illustrates the relation between the absorbance and the V_L_/(V_M_ + V_L_) ratio, where V_M_ and V_L_ denote the volumes of the metal and the ligand, respectively. The findings indicated a break, confirming the 1:1 stoichiometric ratio. The stability constant of formation (K_f_) was calculated through appropriate equation derived from the Beer-Lambert Law and equilibrium expressions related to complex formation^53,54^. The stability constant was determined to be 1.38 × 10^5^ mol^−1^ L. Furthermore, employing the relationship ΔG = − RTlnK_f_, the Gibbs free energy was (ΔG, − 2.88 × 10^4^ kJ mol^−1^) for the metal complex, where R represents the gas constant (8.314 J mol^−1^ K^−1^), T denotes the operating temperature 293.15 K.

**Table 4:** Values of redox potential of H_2_TAP ligand and its complexes.

Compound	Reduction potential (cathode)		Oxidation potential (anode)	
	E_C1_/V (ligand)	E_C2_/V (metal)	E_a1_/V (ligand)	E_a2_/V (metal)
H_2_TAP ligand	− 0.95	–	+ 0.75	–
[Cu(H_2_TAP)(H_2_O)(Cl_2_)]·H_2_O	− 1.10	− 0.45 to − 0.85	+ 0.92	+ 0.06, + 0.31
[Mn(H_2_TAP)(H_2_O)(Cl_2_)]·H_2_O	− 0.93	+ 0.73	+ 0.65	+ 1.00
[Hg(H_2_TAP)(OAc)_2_]·2H_2_O	− 0.94	− 0.55	+ 0.67	+ 0.18

**Table 5:** The maximum wavelength (λ_max_) and Job’s continuous variation method of Hg^II^ complex formation 1 × 10^–3^ mol/L of Hg^II^ and H_2_TAP ligand solution.

a	b			
λ (nm)	Absorbance at [1 M:1L]	[M:L] [1 × 10^–3^ M]	M/L = V_L_/(V_L_ + V_M_)	Absorbance at λmax = 430 nm (pH = 6)
	Hg^II^-H_2_TAP system			Hg^II^-H_2_TAP system
400	1.21	1:9	0.9	0.60
430	1.35	2:8	0.8	1.10
470	1.16	3:7	0.7	1.25
490	1.30	4:6	0.6	1.20
520	0.77	5:5	0.5	1.35
550	0.37	6:4	0.4	0.97
580	0.30	7:3	0.3	0.63
610	0.11	8:2	0.2	0.52
700	0.44	9:1	0.1	0.37
–	–	10:0	0.0	0.0

### Electrochemical properties

#### Cyclic voltammetry study (CV)

Cyclic voltammetry (CV) was employed to investigate the redox behavior of the H₂TAP ligand and its corresponding metal complexes within a potential window of + 1.5 V to − 1.5 V at a scan rate of the 50 mV s^−1^. The voltammogram of the free H₂TAP ligand (Fig. 4a) exhibited a distinct redox couple, characterized by a cathodic peak at Epc = − 0.95 V and an anodic peak at Epa =  + 0.75 V, indicating a quasi-reversible redox process. Upon coordination with the copper metal ion, the voltammogram of the Cu^II^ complex (Fig. 4b) showed a broad cathodic response in the range of − 0.45 V to − 0.85 V, suggestive of two overlapping reduction waves attributed to the Cu^2+^/Cu⁰ couple. This was followed by two anodic peaks at + 0.06 V and + 0.31 V, corresponding to the oxidation of Cu⁰ to Cu^2+^. Coordination also led to notable shifts in the ligand’s original redox peaks, with the reduction potential moving to − 1.10 V and the oxidation peak to + 0.92 V, reflecting altered electronic properties due to metal binding. The Mn^II^ complex (Fig. 4c) displayed a well-defined two-electron quasi-reversible redox process, with a reduction peak at Epc =  + 0.73 V (Mn^2+^ to Mn⁰) and a corresponding oxidation peak at Epa =  + 1.00 V (Mn⁰ to Mn^2+^). Similar to the Cu complex, complexation induced moderate shifts in the ligand’s redox signals (Epc = − 0.93 V and Epa =  + 0.65 V), indicating an electron-rich environment around the metal center. In the case of the Hg^II^ complex (Fig. 4d), two quasi-reversible redox couples were observed. The first reduction event appeared as a weak cathodic peak at Epc = − 0.55 V (Hg^2+^ to Hg⁰), with a corresponding sharp anodic response at Epa =  + 0.18 V. A second, less intense redox couple was noted at Epc = -0.94 V and Epa =  + 0.67 V, as shown in Table 4. These responses also coincided with slight shifts in the redox potentials of the H₂TAP ligand, indicative of moderate interaction and electronic redistribution upon chelation. Overall, the observed redox behavior of the metal complexes confirms successful coordination and highlights the influence of metal ions on the electrochemical properties of the H₂TAP ligand, consistent with the electronic structures proposed from DFT.

#### Correlation with DFT data

These electrochemical findings provide valuable insight into the redox-driven biological activity of the complexes. Notably, the Mn^II^ complex exhibited a well-defined two-electron quasi-reversible redox couple, suggesting an efficient electron-transfer capability, which is a critical feature for radical scavenging and redox-related anticancer mechanisms. This is further supported by its high antioxidant activity (84.1%) and the lowest IC₅₀ value among the tested compounds. The DFT-calculated HOMO–LUMO gap of the Mn^II^ complex (0.1241 eV) is moderate compared to the Cu^II^ and Hg^II^ analogs, indicating sufficient chemical softness and polarizability to engage in biological electron exchange. Furthermore, the Cu^II^ complex, despite exhibiting broader redox features, had a lower antioxidant activity, consistent with its higher electrophilicity index (ω), suggesting stronger reactivity but potentially less biological selectivity. Hence, the quasi-reversible redox behavior observed in the Mn^II^ complex is mechanistically correlated with its favorable electronic profile and pronounced biological performance.

### Gaussian computational studies

#### Molecular modeling

**Fig. 6:**
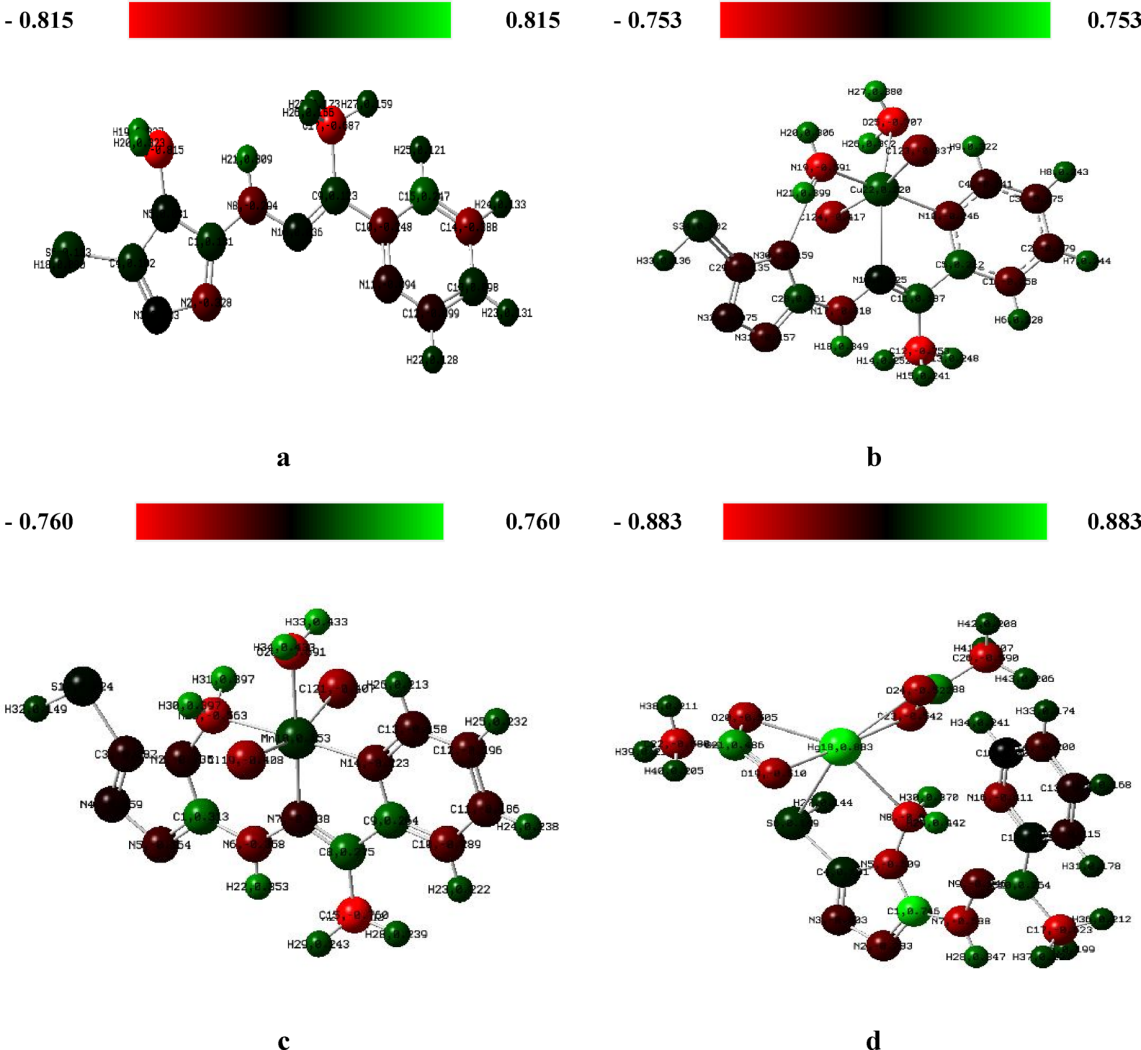
Mulliken charges of )**a**) H_2_TAP ligand, (**b**) [Cu(H_2_TAP)(H_2_O)(Cl_2_)]·H_2_O, (**c**) [Mn(H_2_TAP)(H_2_O)(Cl_2_)]·H_2_O and (**d**) [Hg(H_2_TAP)(OAc)_2_]·2H_2_O complexes.

Density Functional Theory (DFT) calculations were conducted using the mixed basis set (6–311 + G(d,p)/LANL2DZ) to optimize the molecular geometries of the H₂TAP ligand and its metal complexes^55–58^. The optimized structures, as shown in Fig. S8, along with the Mulliken charge distributions (Fig. 6 and Table S7), provide insights into electronic configuration and bonding. Bond length analysis revealed good agreement with experimental IR spectral shifts, supporting the coordination of functional groups to metal centers. Additionally, the bond angles observed for all chelates confirmed distorted octahedral geometries, consistent with the proposed coordination environment^59^, as reported data in Tables 6, S8, and S9 and Figs. S9 and S10.

**Table 6:** The calculated electronic energy, dipole moment and chemical reactivity parameters of )a) H_2_TAP ligand, (b) [Cu(H_2_TAP)(H_2_O)(Cl_2_)]·H_2_O, (c) [Mn(H_2_TAP)(H_2_O)(Cl_2_)]·H_2_O and (d) [Hg(H_2_TAP)(OAc)_2_]·2H_2_O complexes.

Compound	a	b	c	d
Dipole moment (De)	8.0807	5.6913	5.9952	9.8430
Electronic energy (Ha)	− 1131.0	− 1045.3	− 953.12	− 1630.6
E_HOMO_ (eV)	− 0.2153	− 0.2272	− 0.2164	− 0.2392
E_LUMO_ (eV)	− 0.0544	− 0.1729	− 0.0924	− 0.0825
E_gap_ (eV)	0.1609	0.0543	0.1241	0.1566
*χ* (eV)	0.1348	0.2001	0.1544	0.1608
*μ* (eV)	− 0.1348	− 0.2001	− 0.1544	− 0.1608
*η* (eV)	0.0805	0.0272	0.0620	0.0783
*σ* (eV)	12.428	36.832	16.120	12.771
*ω* (eV)	0.1129	0.7372	0.1921	0.1652

#### Molecular chemical parameters

**Fig. 7:**
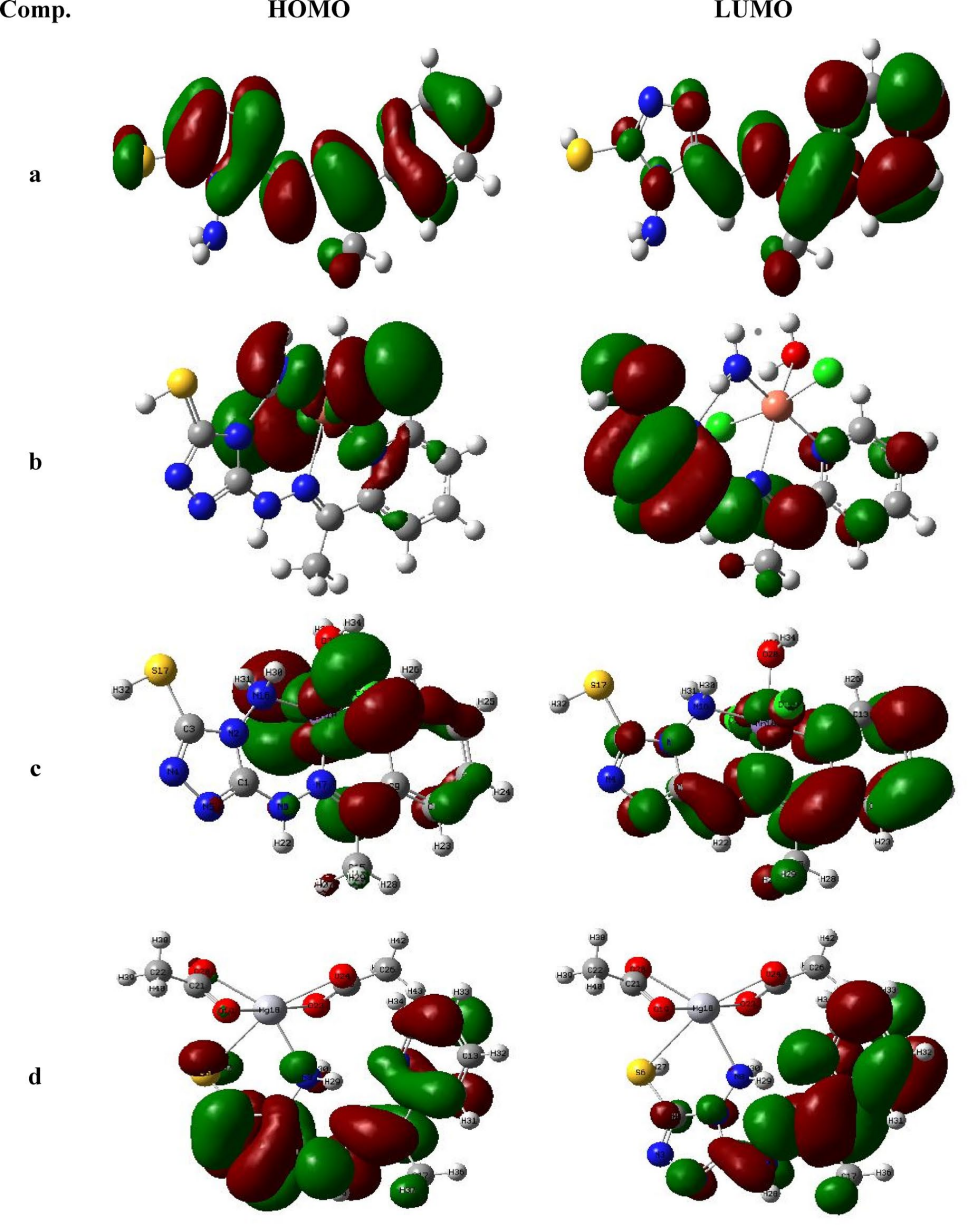
HOMO and LUMO of )**a**) H_2_TAP ligand, (**b**) [Cu(H_2_TAP)(H_2_O)(Cl_2_)]·H_2_O, (**c**) [Mn(H_2_TAP)(H_2_O)(Cl_2_)]·H_2_O and (**d**) [Hg(H_2_TAP)(OAc)_2_]·2H_2_O complexes.

The DFT-derived quantum chemical descriptors, including dipole moments and total electronic energies (E_total_), are summarized in Table 6. Frontier molecular orbital energies (E_HOMO_ and E_LUMO_) were also calculated (Fig. 7), allowing determination of key reactivity indices such as the energy gap (E_gap_), electronegativity (χ), chemical potential (μ), hardness (η), softness (σ)^60^, and electrophilicity index (ω) as presented in the set of Eqs. (6–11). A lower E_gap_ is indicative of enhanced chemical reactivity, polarizability, and potential biological activity.

6 $${\text{E}}_{{{\text{gab}}}} = {\text{ E}}_{{{\text{LUMO}}}} - {\text{ E}}_{{{\text{HOMO}}}}$$

7 $$\chi = \, - \, \left( {{\text{E}}_{{{\text{LUMO}}}} + {\text{E}}_{{{\text{HOMO}}}} } \right)/{2}$$

8 $$\mu = \, - \chi = \left( {{\text{E}}_{{{\text{LUMO}}}} + {\text{ E}}_{{{\text{HOMO}}}} } \right)/{2}$$

9 $$\eta = \left( {{\text{E}}_{{{\text{LUMO}}}} {-}{\text{ E}}_{{{\text{HOMO}}}} } \right) \, /{2}$$

10 $$\upsigma = {1}/\eta^{ - }$$

11 $$\upomega = \mu^{{2}} /{2}\eta$$

Among the studied compounds, the Cu^II^ complex exhibited the highest electronegativity and the most negative chemical potential (μ = − 0.2001 eV), suggesting lower thermodynamic stability and greater chemical reactivity. In contrast, the H₂TAP ligand displayed the highest hardness (η = 0.0805 eV) and lowest softness (σ = 12.428 eV^−1^), attributed to its abundance of electronegative donor atoms (N, O, and S). These characteristics align with the hard-soft acid–base (HSAB) principle, supporting the observed stability of the resulting metal chelates. Based on the electrophilicity index (ω), the compounds follow the order: Cu^II^ complex > Mn^II^ complex > Hg^II^ complex > free ligand, in accordance with their electronic configurations and donor–acceptor interactions^61,62^.

#### Molecular electrostatic potential (MEP)

**Fig. 8:**
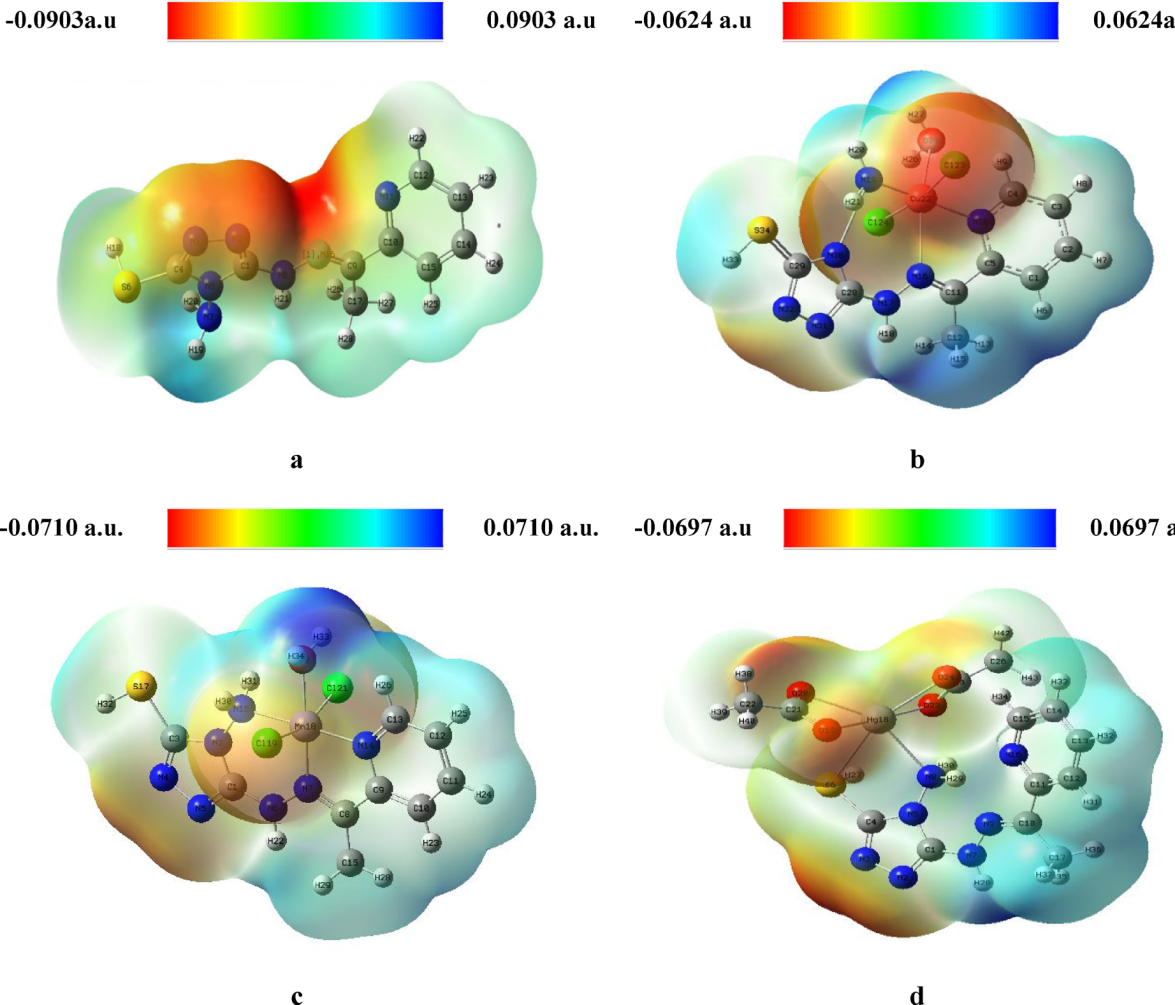
MEP of )**a**) H_2_TAP ligand, (**b**) [Cu(H_2_TAP)(H_2_O)(Cl_2_)]·H_2_O, (**c**) [Mn(H_2_TAP)(H_2_O)(Cl_2_)]·H_2_O and (**d**) [Hg(H_2_TAP)(OAc)_2_]·2H_2_O complexes.

(MEP) to further probe the reactive sites and electron distribution, MEP maps was generated for the ligand and its metal complexes^63^ (Fig. 8). These maps categorize regions of electron density into electrophilic (red), neutral (green), and nucleophilic (blue) zones. The free H_2_TAP ligand displayed pronounced negative electrostatic potential around key electronegative atoms: the triazole nitrogen, thiol sulfur, azomethine nitrogen, and pyridine nitrogen. Conversely, positive potential was primarily localized on hydrogen atoms of the amine (-NH₂) and imine (-NH) groups^64^.

Upon complexation, the Cu^II^ and Hg^II^ species retained regions of negative potential around acetate, coordinated water molecules, and the pyridine ring, indicating possible interaction sites. In contrast, the Mn^II^ complex exhibited reduced negative potential overall, possibly reflecting a more delocalized electron density. These MEP profiles corroborate the coordination modes and support the conclusions drawn from both spectral and quantum chemical analyses.

### Biological investigations

#### Antimicrobial activities

**Fig. 9:**
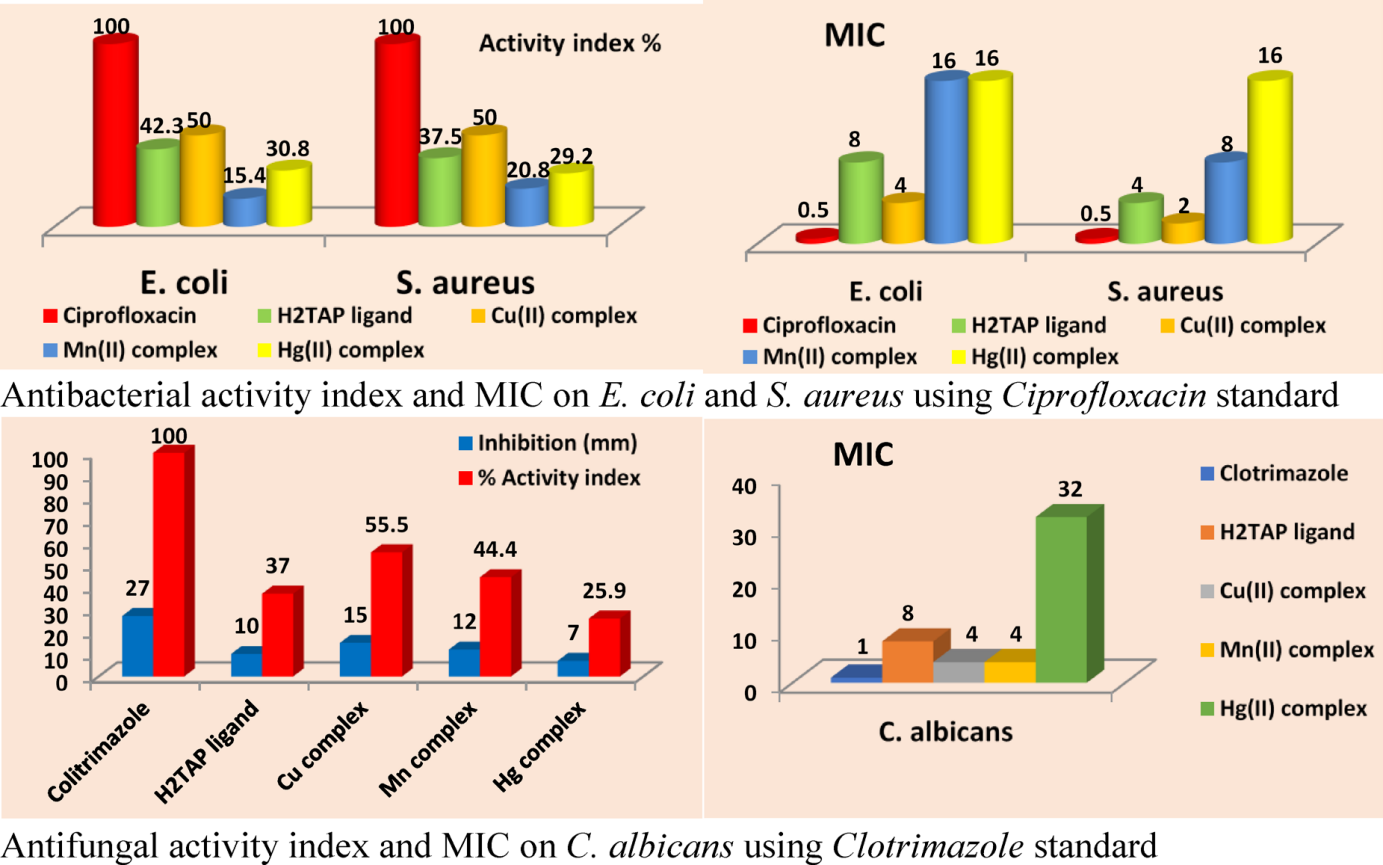
Antimicrobial activity and MIC of isolated solid compounds.

The antimicrobial activities of the H₂TAP ligand and its metal complexes were evaluated in vitro against *Escherichia coli* (Gram-negative), *Staphylococcus aureus* (Gram-positive), and *Candida albicans* (fungus). The percentage activity index was calculated based on inhibition zone diameters using the equation %AI = (Inhibition zone of the sample (mm) / Inhibition zone of the standard (mm)) × 100^65,66^, as described in Table S10. Compared to the reference drug *Ciprofloxacin*, the tested compounds demonstrated variable antibacterial efficacy, following the order Cu^II^ > H₂TAP > Mn^II^ > Hg^II^. The activity against both *E. coli* and *S. aureus* was generally modest to minimal. In contrast, the antifungal evaluation against *C. albicans* (relative to *Clotrimazole*) revealed notable activity for all tested compounds. The antifungal potency followed the trend Cu^II^ > Mn^II^ > H₂TAP > Hg^II^, as illustrated in Fig. 9. The MIC values further supported this trend, with the Cu^II^ complex exhibiting the lowest MICs against against *E. coli* (4 µg/mL) and *S. aureus* (2 µg/mL), and also *C. albicans* (4 µg/mL), whereas the Hg^II^ complex showed the weakest activity (16 µg/mL) for both bacterial strains and (32 µg/mL) for the fungal organism.

These observations can be rationalized based on coordination chemistry principles. The coordination behavior of the H₂TAP ligand significantly varies depending on the metal center, which directly impacts the antimicrobial performance of the resulting complexes. Both Cu^II^ and Mn^II^ exhibit tridentate coordination through amine, imine, and pyridine nitrogen atoms, forming fused five- and six-membered chelate rings. This structural arrangement enhances complex stability and facilitates microbial membrane penetration. In contrast, the Hg^II^ complex coordinates via only two donor atoms (-NH₂ and -SH), forming a single five-membered ring, which results in lower stability and reduced biological activity. These findings indicate that the antimicrobial efficacy is governed by the combined effect of the metal’s intrinsic properties such as redox activity and biological relevance and the ligand’s coordination mode and chelation geometry. Accordingly, the superior potency of the Cu^II^ complex is attributed to its favorable coordination structure and redox-active nature, while the weak performance of the Hg^II^ complex stems from limited chelation and weaker metal–ligand interaction.

#### ABTS antioxidant

**Fig. 10:**
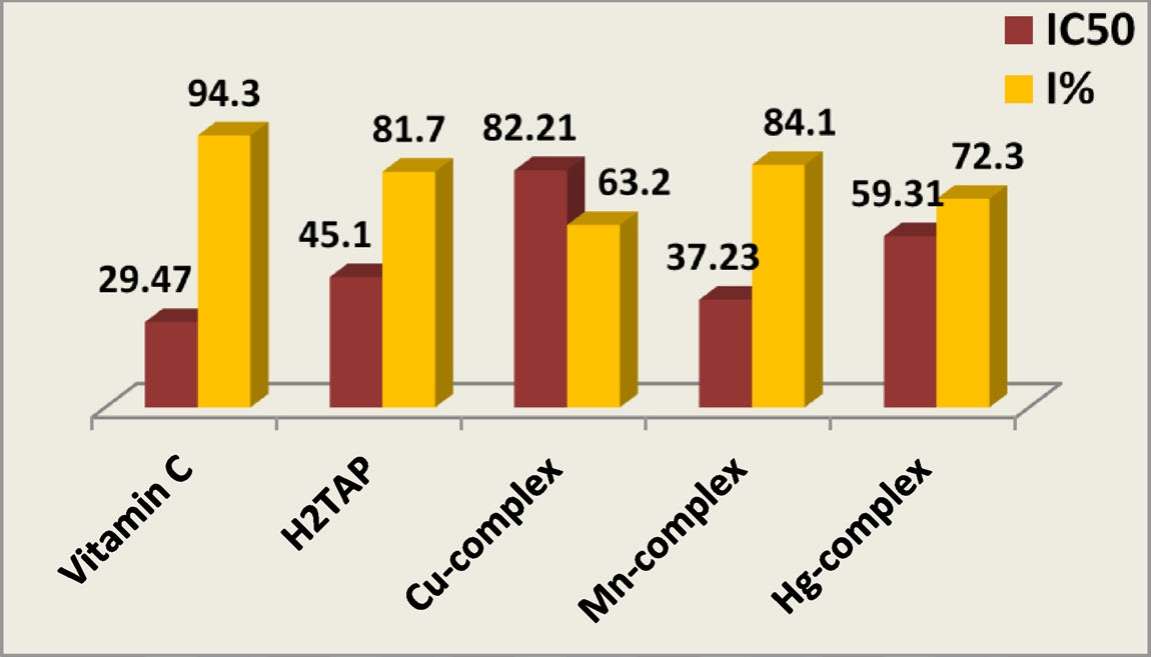
Inhibition effect (I%) and IC_50_ for the antioxidant ABTS method using *Vitamin C* standard.

**Fig. 11:**
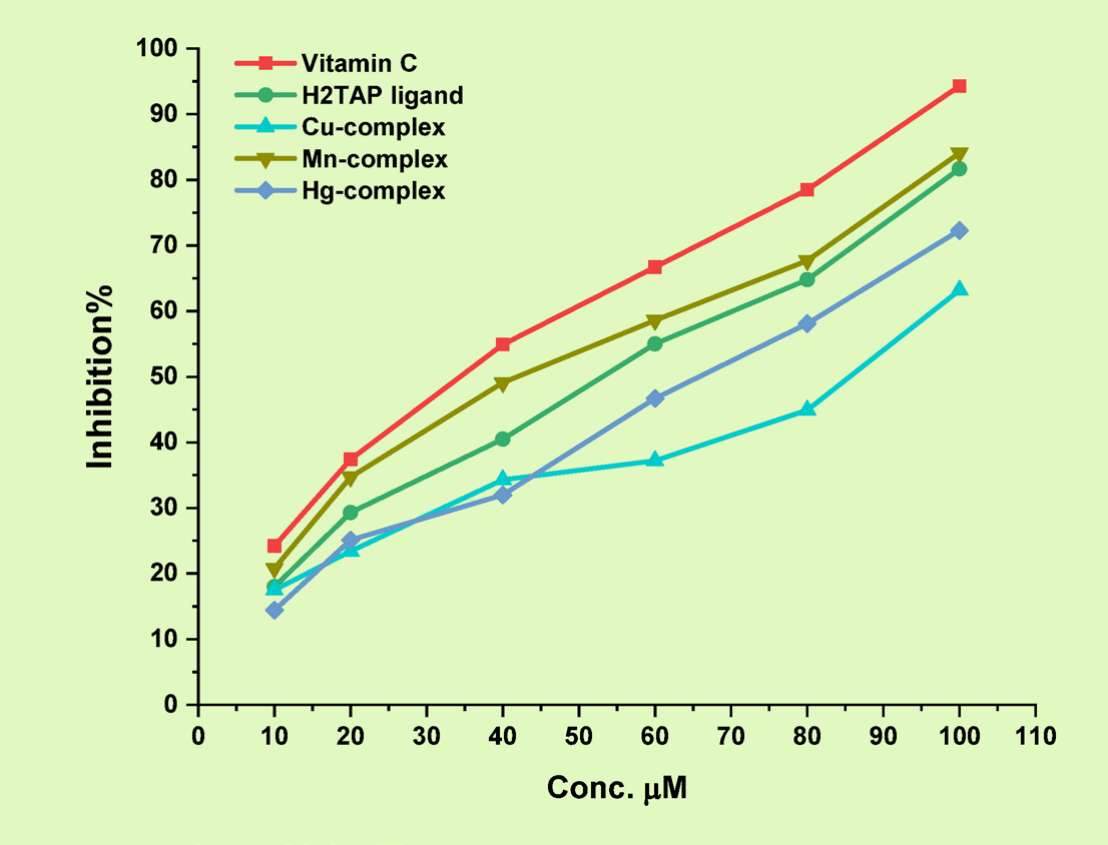
The plot of Conc. *vs.* Inhibition% of ABTS method.

The antioxidant potential of the H₂TAP ligand and its metal complexes was assessed using the ABTS radical scavenging assay at a fixed concentration of 100 µM. *Vitamin C* was employed as a positive control for comparative evaluation^67,68^. The percentage inhibition was calculated using the standard formula: % Inhibitory impact (I) = [(Absorbance of control—Absorbance of sample)/Absorbance of control] × 100. Based on the measured inhibition values, the compounds demonstrated varying degrees of antioxidant activity, following the order Mn^II^ complex (84.1%) > H₂TAP ligand (81.7%) > Hg^II^ complex (72.3%) > Cu^II^ complex (63.2%), as illustrated in Fig. 10. To further quantify antioxidant efficiency, IC₅₀ values were determined through serial dilutions (100, 80, 60, 40, 20, and 10 µM). The resulting data were plotted as inhibition percentage versus concentration, and the IC₅₀ values were derived accordingly (Fig. 11 and Table S11). These findings confirm that the Mn^II^ complex exhibits the most potent radical scavenging capability among the tested compounds.

#### The cytotoxicity

**Fig. 12:**
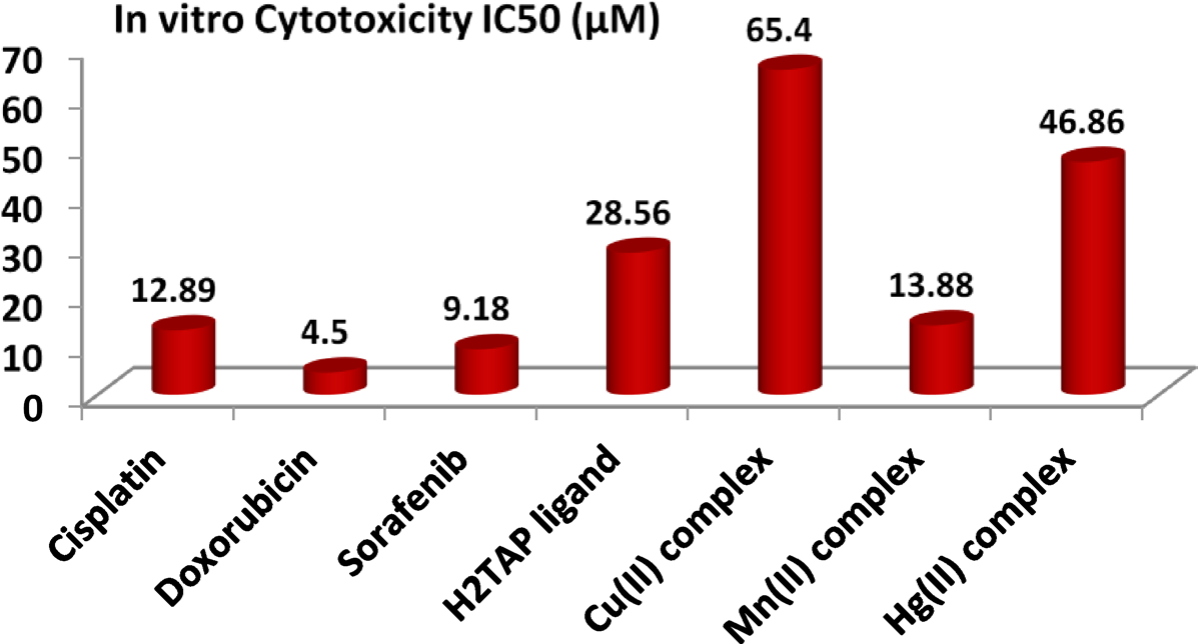
Cytotoxicity IC_50_ (µM) of the ligand and its metal complexes.

**Fig. 13:**
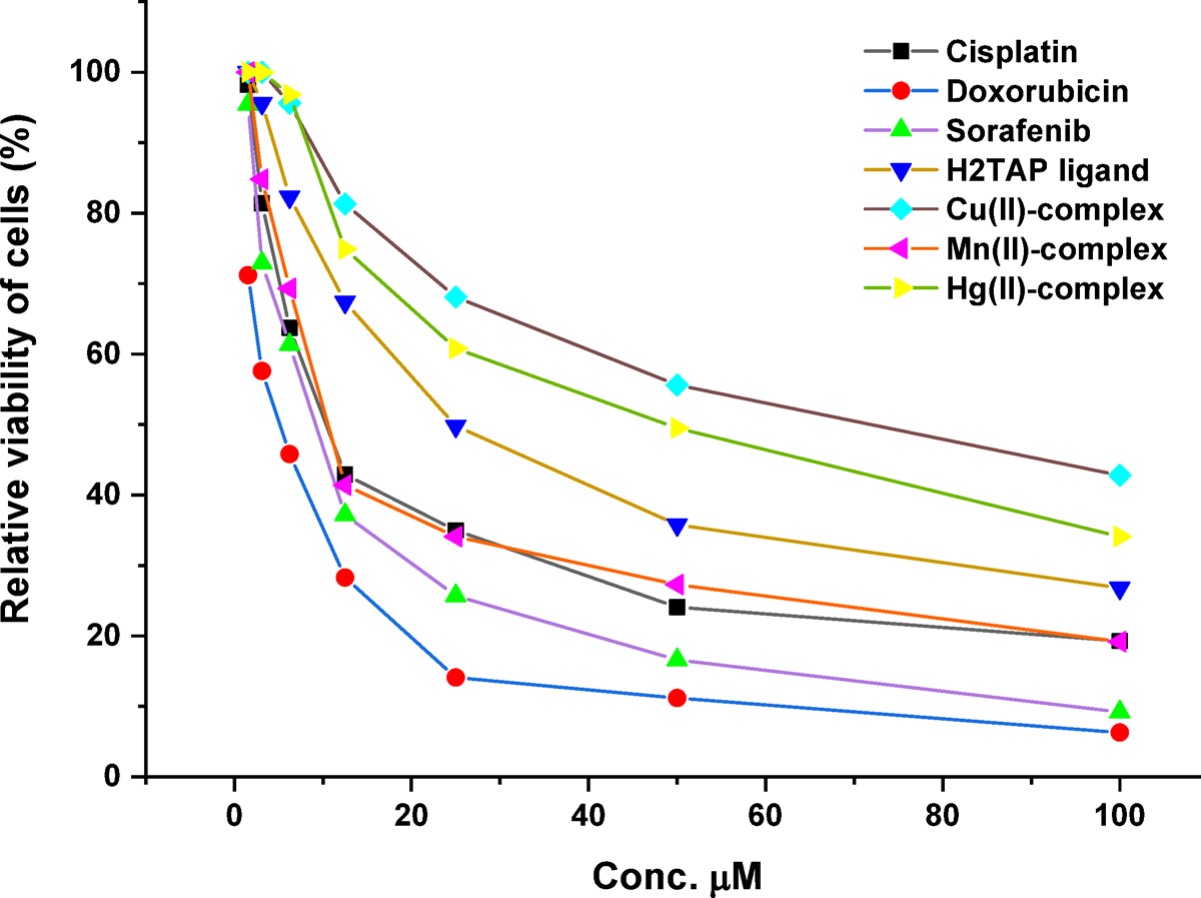
The plot of series dilutions versus cell viability % in cell line HePG-2.

The synthesized ligand and its chelates were assessed by the MTT assay on HePG-2 human hepatocellular carcinoma cells, utilizing *Cis-platin*, *Doxorubicin,* and *Sorafenib* as positive references. This test depends on the conversion of tetrazolium bromide (MTT-yellow) into a purple formazan derivative via mitochondrial succinate dehydrogenase in live cells, which may be measured using spectrophotometry. The cell viability percentage is calculated by applying the relation % Cell viability = (Mean absorbance of treated cells/Mean absorbance of untreated cells) × 100^69–71^. At determination of the IC_50_, cells were treated with successive dilutions (100, 50, 25, 12.5, 6.25, 3.125, 1.5625 µM) of the substances under investigation. The concentrations of each component (Conc.) were plotted against (% Cell Viability) using GraphPad Prism 9 software, as shown in Figs. 12 and 13 and detailed in Table S12.

The results suggest that the studied compounds may be evaluated in the following manner: (**i**) All substances display highly hazardous compounds with low IC_50_ values; the H_2_TAP ligand and Mn^II^ complex demonstrate the greatest compatibility with *Cis-platin* standard; (**ii**) The Mn^II^ complex exhibited the lowest IC₅₀ value (13.88 ± 1.2 µM) against HepG2 cells among all tested compounds, indicating strong cytotoxic potential. This biological behavior can be rationalized by its moderate electrophilicity index (ω) and balanced HOMO–LUMO gap, which together imply an optimal reactivity profile for selective biological interactions. The moderate energy gap facilitates electron transfer processes essential for disrupting redox homeostasis in cancer cells while maintaining sufficient molecular stability; (**iii**) In addition, although the Cu^II^ complex shows a more negative chemical potential (μ = − 0.2001 eV) and higher electrophilicity, its biological effect was comparatively weaker (IC₅₀ = 65.40 ± 3.6 µM), likely due to excessive reactivity that could compromise selectivity or cellular uptake. On the other hand, the Mn^II^ complex presents a favorable balance, with moderate electronegativity and a more adaptable redox character; (**iv**) Structurally, the Mn^II^ center adopts a distorted octahedral geometry, providing flexible coordination that may enhance interactions with biological targets such as DNA, enzymes, or mitochondrial components. This geometry, combined with the ligand’s electron-rich donor atoms (N, S, and O), likely contributes to efficient cellular penetration and interaction with vital intracellular biomolecules. Thus, the observed cytotoxicity of the Mn^II^ complex is mechanistically consistent with its electronic descriptors and coordination flexibility, supporting its potential as a redox-active anticancer agent.

#### DNA-binding

**Fig. 14:**
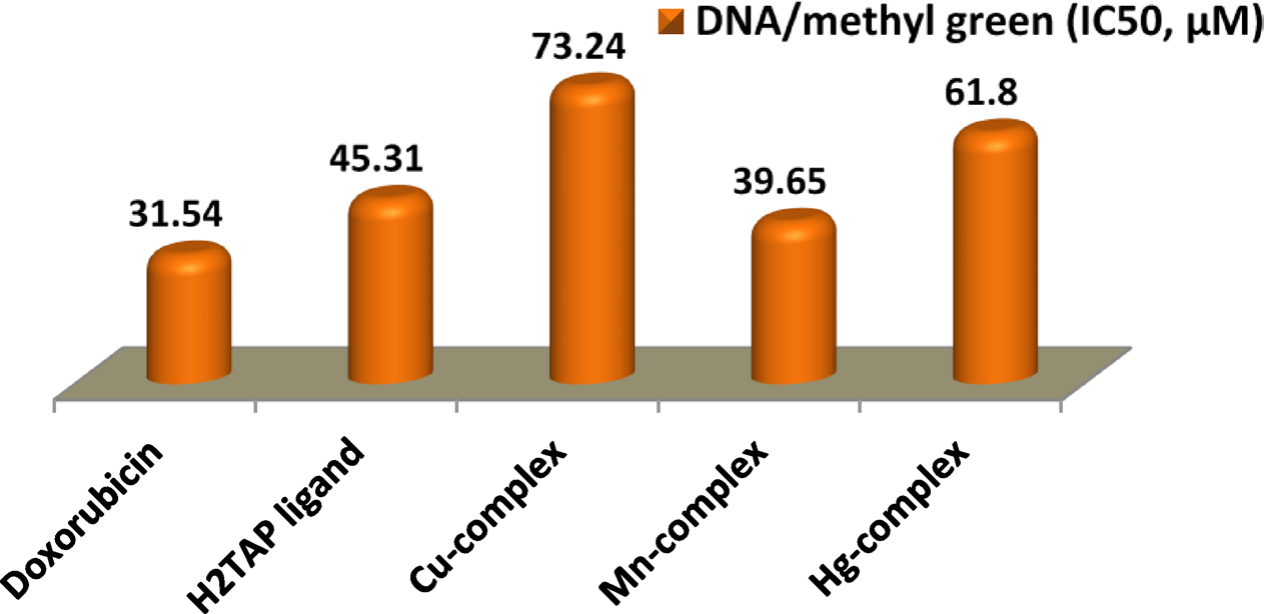
DNA binding assay for prepared compounds.

Schiff bases are characterized by the presence of an azomethine group (–C=N–), which facilitates the formation of stable metal complexes. This structural feature enhances their potential to interact with DNA, making Schiff base metal chelates promising candidates for anticancer therapy. DNA-binding affinity was evaluated using the methyl green (MG) displacement assay. MG is a DNA-intercalating dye, and its absorbance decreases upon displacement by compounds with higher DNA affinity. A significant reduction in MG absorbance therefore indicates effective DNA binding by the test compounds. The results, summarized in Table S13 and illustrated in Fig. 14, revealed that the Mn^II^ complex exhibited the highest DNA-binding affinity, as indicated by the lowest IC₅₀ value defined as the concentration required to reduce the initial absorbance of the DNA-MG complex by 50%. The other tested compounds showed moderate to low DNA-binding activity in comparison to the standard drug *Doxorubicin*.

#### The molecular docking

**Fig. 15:**
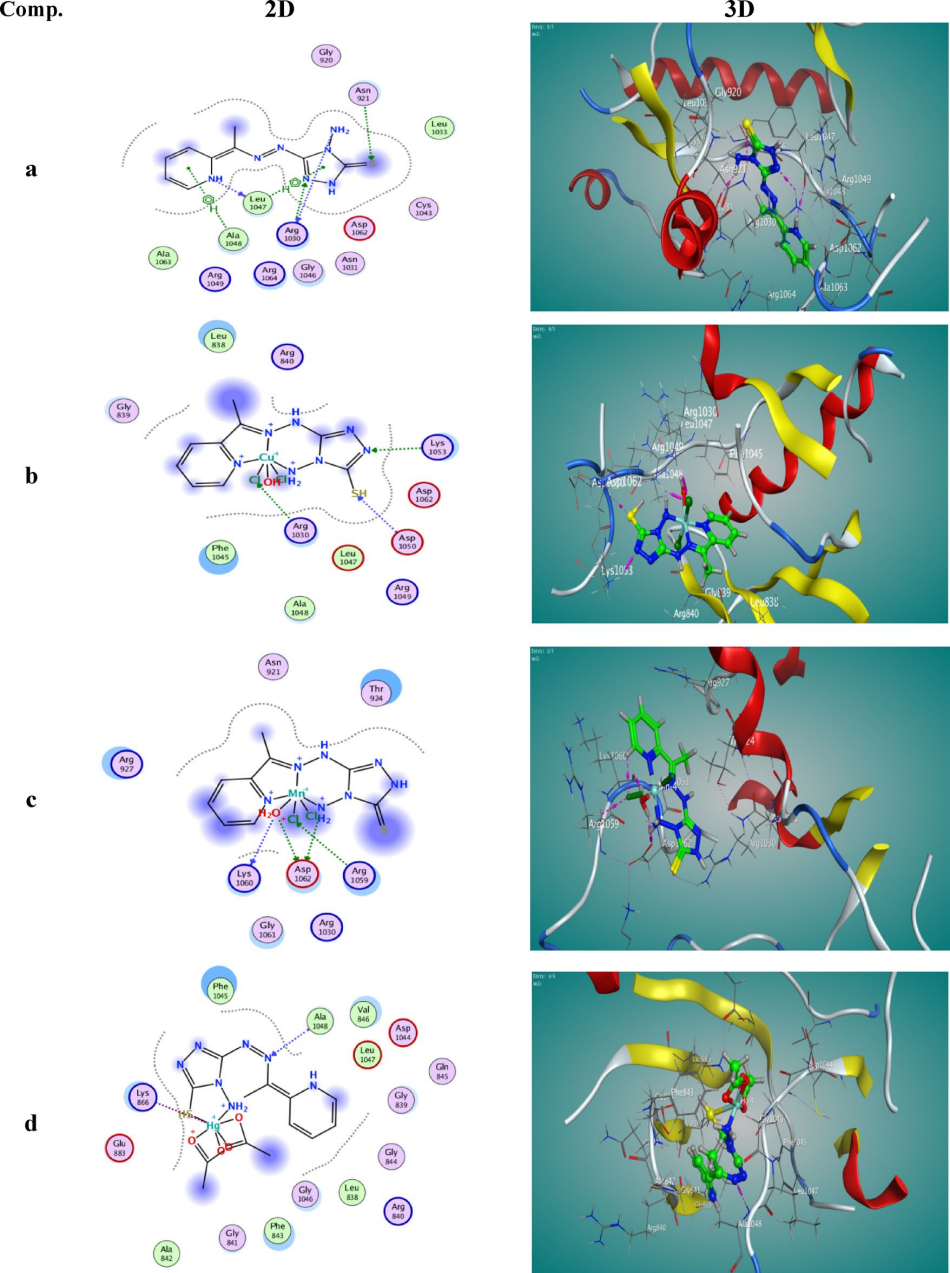
2D and 3D binding poses obtained from docking studies between )**a**) H_2_TAP ligand, (**b**) [Cu(H_2_TAP)(H_2_O)(Cl_2_)]·H_2_O, (**c**) [Mn(H_2_TAP)(H_2_O)(Cl_2_)]·H_2_O and (**d**) [Hg(H_2_TAP)(OAc)_2_]·2H_2_O complexes with human hepatocellular cancer with (PDB: 1YWN).

**Fig. 16:**
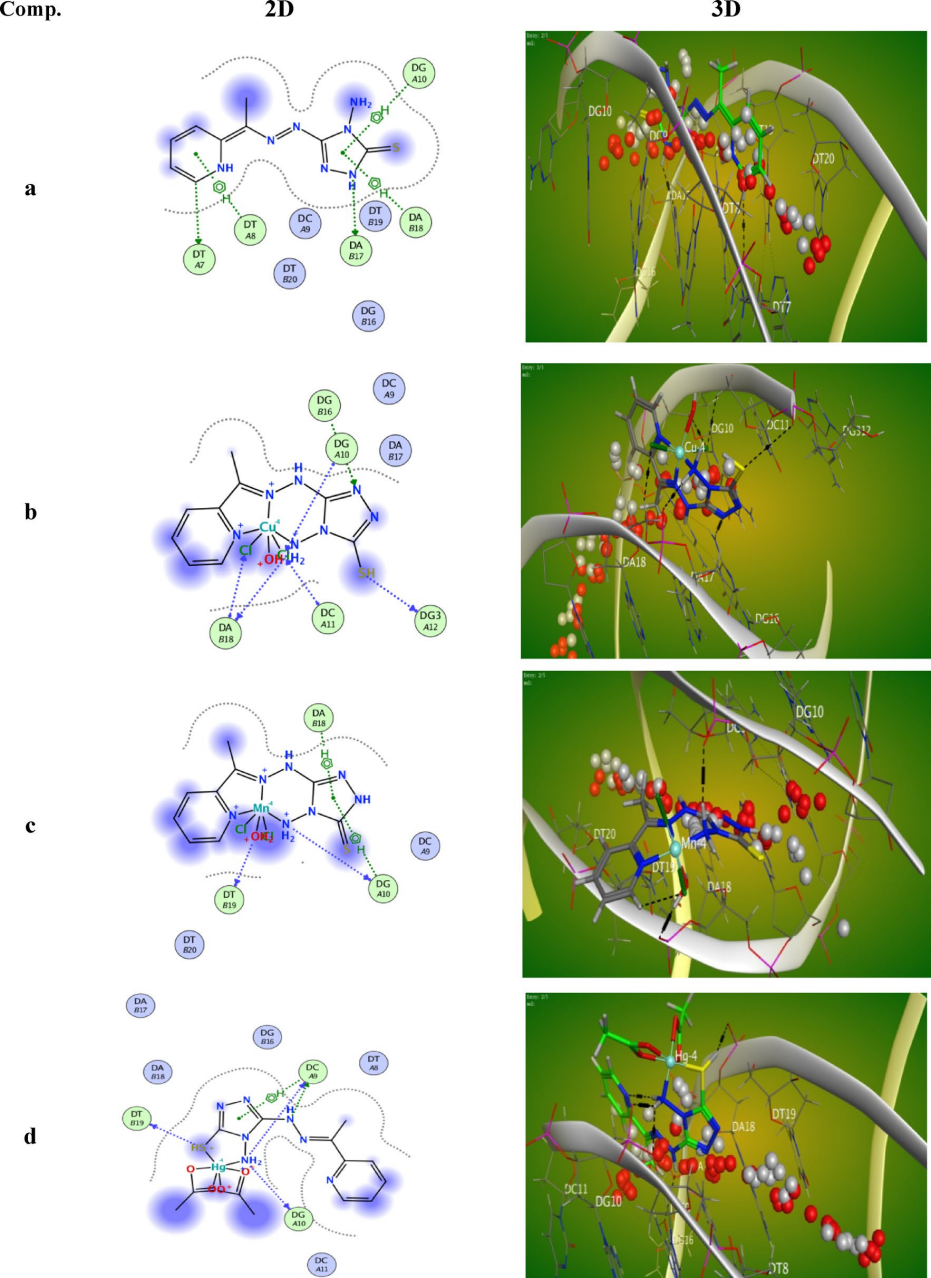
2D and 3D binding poses obtained from docking studies between )**a**) H_2_TAP ligand, (**b**) [Cu(H_2_TAP)(H_2_O)(Cl_2_)]·H_2_O, (**c**) [Mn(H_2_TAP)(H_2_O)(Cl_2_)]·H_2_O and (**d**) [Hg(H_2_TAP)(OAc)_2_]·2H_2_O complexes with DNA with (PDB: 8EC1).

Molecular docking is a widely used computational technique in drug design, aimed at simulating molecular recognition by predicting the optimal binding conformation between a ligand and a target receptor, thereby minimizing the system’s free energy^72–75^. In this study, molecular docking was performed for the H₂TAP ligand and its metal complexes with two targets: human hepatocellular carcinoma protein (PDB ID: 1YWN) and DNA (PDB ID: 8EC1). The results demonstrated significant interactions between the tested compounds and the selected receptors, as illustrated in Figs. 15 and 16. The corresponding binding energies and interaction parameters are summarized in Table 7. Both 2D and 3D visualizations confirmed the localization of the ligands within the active sites of the receptors. Compounds exhibiting more negative binding free energy values were considered to have stronger binding affinities. Among the tested compounds, the H₂TAP ligand showed the highest binding affinity with the lowest (most negative) estimated free energy of binding. The order of binding affinity with the 1YWN protein was H₂TAP > Mn^II^ complex > Cu^II^ complex > Hg^II^ complex, whereas for the 8EC1 DNA target, the order was H₂TAP > Hg^II^ complex > Cu^II^ complex > Mn^II^ complex.

**Table 7:** All docking results and interactions between (a) H_2_TAP ligand, (b) [Cu(H_2_TAP)(H_2_O)Cl_2_].H_2_O, (c) [Mn(H_2_TAP)(H_2_O)Cl_2_].H_2_O and (**d**) [Hg(H_2_TAP)(OAc)_2_].2H_2_O complexes and target enzymes of human hepatocellular cancer cell lines (PDB: 1YWN) and DNA (PDB: 8EC1).

Comp	(PDB: code)	S	RMSD	E score	Number of bonds	Interaction types	Distance (A°)	Energy (kcal/mol)
a	1YWN	− 5.6504	1.3322	− 8.6219	N(9)–ARG(1030)	H-donor	2.84	− 1.3
					N(23)–LEU(1047)	H-donor	3.06	− 0.8
					N(2)–ARG(1030)	H-acceptor	3.04	− 1.2
					S(7)–ASN(921)	H-acceptor	4.28	− 0.7
					5-ring–LEU(1047)	π–H	3.24	− 1.5
					6-ring–ALA(1048)	π–H	4.18	− 1.3
	8EC1	− 6.5087	1.6893	− 8.9969	N(3)–DA(17)	H-donor	3.18	− 2.0
					C(21)–DT(7)	H-donor	3.37	− 0.8
					6-ring–DT(8)	π–H	4.17	− 0.7
					5-ring–DG(10)	π–H	4.69	− 0.6
					5-ring–DA(18)	π–H	3.44	− 1.0
b	1YWN	− 4.1970	1.6132	− 12.541	N(4)–LYS(1053)	H-acceptor	3.11	− 6.8
					S(30)–ASP(1050)	H-acceptor	3.72	− 1.1
					Cl(32)–ARG(1030)	H-acceptor	3.25	− 7.5
					Cl(32)–ARG(1030)	H-acceptor	3.15	− 8.6
	8EC1	− 5.2411	3.5952	− 12.162	N(27)–DA(18)	H-donor	3.54	− 0.9
					S(30)–DG3(12)	H-donor	3.62	− 1.2
					N(5)–DG(16)	H-acceptor	3.01	− 4.0
					C(32)–DA(18)	H-acceptor	3.45	− 1.6
					C(33)–DG(10)	H-acceptor	3.24	− 1.5
					C(33)–DC(11)	H-acceptor	3.30	− 1.1
c	1YWN	− 4.9634	2.4949	− 8.5929	N(25)–ASP(1062)	H-donor	2.89	− 3.6
					O(31)–LYS(1060)	H-donor	2.81	− 2.4
					O(31)–ASP(1062)	H-donor	2.70	− 2.4
					Cl(34)–ARG(1059)	H-acceptor	3.57	− 1.4
	8EC1	− 4.9438	2.5614	− 12.406	N(25)–DG(10)	H-donor	3.26	− 3.2
					O(31)–DT(19)	H-donor	2.73	− 4.1
					5-ring–DG(10)	H–π	3.55	− 0.7
					5-ring–DA(18)	H–π	4.00	− 0.7
d	1YWN	15.967	1.9092	− 8.4536	N(12)–ALA(1048)	H-acceptor	3.07	− 4.9
					Hg(29)–LYS(866)	ionic	2.73	− 6.6
	8EC1	− 5.8234	2.1466	− 9.7535	S(6)–T(19)	H-donor	4.00	− 1.9
					N(8)–DC(9)	H-donor	3.24	− 0.9
					N(10)–DC(9)	H-donor	3.21	− 0.8
					N(10)–DG(10)	H-donor	3.25	− 4.5
					5-ring–DC(9)	H–π	3.58	− 1.1

#### The structure–activity relationship studies (SAR)

The structure–activity relationship (SAR) analysis was conducted based on density functional theory (DFT) calculations, particularly focusing on key molecular descriptors such as the HOMO–LUMO energy gap and dipole moment. These electronic parameters play a vital role in predicting membrane permeability and drug excretion rates and can be correlated with the compounds’ antimicrobial and antioxidant properties^76^. The SAR findings revealed an inverse relationship between the dipole moment and antimicrobial efficacy. A lower dipole moment is associated with increased lipophilicity, which enhances the ability of a compound to penetrate microbial lipid membranes, thereby improving its biological effectiveness. Among the tested compounds, the Cu^II^ complex exhibited the lowest dipole moment, which aligns with its superior antimicrobial activity. In contrast, the higher dipole moments observed for the free H₂TAP ligand, Mn^II^, and Hg^II^ complexes are consistent with their comparatively reduced antimicrobial performance. This trend suggests that increased dipole moments hinder the compounds’ ability to traverse the lipid bilayers of microbial cells, thereby limiting their biological activity^77,78^.

#### Drug-likeness prediction

The H_2_TAP ligand and its chelates were assessed for adherence to the Lipinski rule of five, which dictates that an orally active pharmaceutical must not surpass one infraction of the following criteria: hydrogen bond donors (HBD: total number of H–O and H-N bonds) ≤ 5 hydrogen bond acceptors (HBA: total number of O or N atoms) ≤ 10, octanol–water partition coefficient (logP) ≤ 5, and molecular weight (M.wt) ≤ 500^79,80^. The molecular weight substantially influences medications that interact with drug receptors or DNA. As molecular weight increases, the density of the molecules also rises. In addition, logP results have a much bigger effect on improving permeability and inactive membrane division than they do on solubility. Ultimately, polar surface atoms (PSA), such as oxygen, nitrogen, and sulfur, are essential for evaluating drug transport properties. MolSoft software was used to make a model for judging the drug-likeness score of the compounds that were looked at and their molecular features, which can be seen in Fig. S11 and Table 8. Most of the compounds have HBD and HBA values that meet the requirements. This means that the compounds that are made are likely to be able to pass through cell membranes and work as medicines.

**Table 8:** The calculated parameters based on the Lipinski rule.

Compound	M.wt	logP	HBA	HBD	PSA	M. volume
H_2_TAP	249.30	0.45	7	3	94.02	209.52
[Cu(H_2_TAP)(Cl_2_)(H_2_O)].H_2_O	419.77	1.04	7	3	75.90	279.31
[Mn(H_2_TAP)(Cl_2_)(H_2_O)].H_2_O	411.16	1.43	7	3	75.90	279.31
[Hg(H_2_TAP)(OAc)_2_].2H_2_O	604.00	− 2.85	10	2	114.85	323.86

## Conclusion

The CHN analysis and physical features corroborate the proposed chemical formulas of Cu^II^, Mn^II^, and Hg^II^ chelates. The synthesis of complexes and the attachment of chloride/acetate anions to the central metal ion demonstrate the coordinating characteristics of the complexes. The investigations revealed that the ligand functioned as a neutral bidentate or tridentate chelating agent. All complexes exhibited an octahedral configuration. The ^1^H-NMR and ^13^C-NMR spectra of H_2_TAP elucidate chemical shifts and assignments, whilst the spectra of the Hg^II^ complex give further characterization. The TGA/DTA study offered insights into the thermal degradation of the compounds. PXRD investigation revealed the polycrystalline characteristics of the Mn^II^ and Cu^II^ complexes. The CV analysis indicated a robust interaction between the ligand and the mercury species in the solution measurements. The formation constants (βj) derived from the Job’s approach indicated a favorable ligand–metal stoichiometry of (1L:1M). The H_2_TAP ligand and its metal chelates have interesting biological activities, exhibiting modest antibacterial, substantial antifungal, and medium-strong cytotoxicity effects. Moreover, they exhibited diverse antioxidant activities and satisfactory affinity for DNA. Molecular docking demonstrated several intermolecular interactions between the protein receptors and manufactured complexes, confirming their efficacy. The examined compounds comply with the Lipinski criterion, suggesting their potential as orally active pharmaceuticals.

## Supplementary Information

Supplementary Information.

## Data Availability

The data supporting the research findings can be obtained from the corresponding author upon reasonable request.

## References

[1] Li, C., Yu, K., Yang, K. & Song, Q. Nickel-catalyzed reductive difunctionalization of BN-heterocyclic olefins. *Org. Chem. Front.* **12**, 1162–1166. 10.1039/D4QO02032D (2025).

[2] Aufricht, P. *et al.* Formic acid dehydrogenation catalysed by a novel amino-di(N-heterocyclic carbene) based Ru-CNC pincer complex. *Chem. Commun.* **61**. 3923. 10.1039/d4cc05164e (2025). 10.1039/d4cc05164e 39820207

[3] Muzaffar, S. *et al.* Heteroleptic Zn(II) complexes; synthesis, spectral characterization, DNA interaction, enzyme inhibition and docking studies. *J. Mol. Struct.* **1326**, 141078. 10.1016/j.molstruc.2024.141078 (2025).

[4] Mohanty, M. *et al.* RuIII–morpholine-derived thiosemicarbazone-based metallodrugs: Lysosome-targeted anticancer agents. *ACS Appl. Bio Mater.*10.1021/acsabm.4c01536 (2025). 39806879 10.1021/acsabm.4c01536

[5] Manisha, *et al.* 3d-transition metal complexes of a tridentate ligand: Synthesis, characterization, physico-chemical studies, antimicrobial activity, in silico molecular docking and ADME studies. *Chem. Africa.*10.1007/s42250-025-01310-3 (2025).

[6] Alka, *et al.* New Development of triaminepyrimidine derived tridentate Schiff’s base ligand and its Mn (II), Co (II), Ni (II), Cu (II) complexes: Synthesis, spectral, molecular docking, DFT, in silico ADMET and biological screening. *Appl. Organomet. Chem.* **39**, e70076. 10.1002/aoc.70076 (2025).

[7] Verma, C. *et al.* Coordination bonding and corrosion inhibition potential of nitrogen-rich heterocycles: Azoles and triazines as specific examples. *Coord. Chem. Rev.* **488**, 215177. 10.1016/j.ccr.2023.215177 (2023).

[8] Wang, J. *et al.* Frontiers and advances in N-heterocycle compounds as corrosion inhibitors in acid medium: Recent advances. *Adv. Colloid Interface Sci.* **321**, 10.1016/j.cis.2023.103031 (2023). 10.1016/j.cis.2023.103031 37907032

[9] Alka, Singh, J., Kumari, P. & Jain, P. Synthesis, characterization, biological, ADMET, and molecular docking studies of transition metal complexes of aminopyridine Schiff base derivative. *Chem. Biodivers.* **21**, e202401101. 10.1002/cbdv.202401101 (2024).

[10] Zhao, J. *et al.* Heterocyclic molecules tethered branched polymers with innate immune stimulating activity. *Ccs Chem.* **6**, 1278–1288. 10.31635/ccschem.023.202303214 (2024).

[11] Mezgebe, K., Melaku, Y., Ramachandran, V. P. & Mulugeta, E. Synthesis, dyeing performance and evaluation of the antimicrobial and antioxidant activities of azo dye derivatives incorporated with 1, 3, 4-thiadiazole combined with in silico computational studies. *New J. Chem.* **48**, 4400–4416. 10.1039/D3NJ04790C (2024).

[12] Wang, W. *et al.* TFA-mediated nitrogenous heterocyclic assisted aldimine condensation/cyclization for the synthesis of pyrrolo [2, 1: 3, 4] quinoxalino [1, 2-c] quinazoline derivatives. *Mol. Catal.* **572**, 114733. 10.1016/j.mcat.2024.114733 (2025).

[13] Race, J. J., Hudson, L. A. & Albrecht, M. Stable CAAC-triazenes: A new nitrogen ligand system with donor and conformational flexibility, and with application in olefin activation catalysis. *Chem. Eur. J.* **30**, e202400400. 10.1002/chem.202400400 (2024). 38687878 10.1002/chem.202400400

[14] Kumar, R. *et al.* Recent advances in synthesis of heterocyclic Schiff base transition metal complexes and their antimicrobial activities especially antibacterial and antifungal. *J. Mol. Struct.* **1294**, 136346. 10.1016/j.molstruc.2023.136346 (2023).

[15] Vaishya, V., Patider, S. & Pilania, M. Imidazolium/triazolium based NHC–palladium complexes and their application in catalysis. *Mater. Today Proc.* **43**, 3181–3187. 10.1016/j.matpr.2021.01.665 (2021).

[16] Manisha *et al.* Synthesis, characterization, biological activity, DFT, molecular docking and ADME studies of metal (II) complexes of a bidentate Schiff’s base (E)-4-chloro-2-((2-hydroxy-3-methoxybenzylidene) amino) benzoic acid. *J. Dispers. Sci. Technol.* 1–14. 10.1080/01932691.2025.2461124 (2025).

[17] Jain, P. *et al.* Bioactive thiosemicarbazone coordination metal complexes: synthesis, characterization, theoretical analysis, biological activity, molecular docking and ADME analysis. *Chem. Biodivers.* **20**, e202300760. 10.1002/cbdv.202300760 (2023). 37427893 10.1002/cbdv.202300760

[18] Jain, P., Kumar, D., Chandra, S. & Misra, N. Experimental and theoretical studies of Mn(II) and Co(II) metal complexes of a tridentate Schiff’s base ligand and their biological activities. *Appl. Organomet. Chem.* **34**, 1–18. 10.1002/aoc.5371 (2020).

[19] Poland, E. M. & Ho, C. C. Photoactive N-heterocyclic carbene transition metal complexes in bond-forming photocatalysis: State-of-the-art and opportunities. *Appl. Organomet. Chem.* **38**, e6746. 10.1002/aoc.6746 (2024).

[20] Li, H., Zhang, L., Li, X., Wang, Y.-S. & Han, Y.-F. Stimulus-responsive organometallic assemblies based on azobenzene-functionalized Poly-NHC ligands. *Chem. Asian J.* e202401421. 10.1002/asia.202401421 (2025). 10.1002/asia.202401421 39777425

[21] Kumar, A., James, G., Aparna, R. K. & Mandal, S. Rational design and synthesis of atomically precise nanocluster-based nanocomposites: a step towards environmental catalysis. *Chem. Commun.* **61**, 2723–2741. 10.1039/D4CC05255B (2025). 10.1039/d4cc05255b 39813088

[22] Sadowska, P., Jankowski, W., Bregier-Jarzębowska, R., Pietrzyk, P. & Jastrząb, R. Deciphering the impact of nucleosides and nucleotides on copper ion and dopamine coordination dynamics. *Int. J. Mol. Sci.* **25**, 10.3390/ijms25179137 (2024). 10.3390/ijms25179137 PMC11394690 39273086

[23] Samy, M. S., Abou El Nadar, H. M., Gomaa, E. A. & Abd El-Hady, M. N. Spectral, DFT, thermal, electrochemical, and biological investigations on Cu^2+^, Cd^2+^, and Mn^2+^ complexes of favipiravir ligand. *Inorg. Chem. Commun.* **150**, 110466. 10.1016/j.inoche.2023.110466 (2023).

[24] Huang, T., Du, P., Cheng, X. & Lin, Y.-M. Manganese complexes with consecutive Mn (IV)? Mn (III) excitation for versatile photoredox catalysis. *J. Am. Chem. Soc.* **146**, 24515–24525. 10.1021/jacs.4c07084 (2024). 39079011 10.1021/jacs.4c07084

[25] Morsali, A. & Masoomi, M. Y. Structures and properties of mercury(II) coordination polymers. *Coord. Chem. Rev.* **253**, 1882–1905. 10.1016/j.ccr.2009.02.018 (2009).

[26] Peng, Z. *et al.* Synthesis, antioxidant and anti-tyrosinase activity of 1,2,4-triazole hydrazones as antibrowning agents. *Food Chem.* **341**, 10.1016/j.foodchem.2020.128265 (2021). 10.1016/j.foodchem.2020.128265 33031957

[27] Feris, E. J., Michael, D., COLE, international application published under the patent cooperation treaty (PCT), WO **113347** Al. (2021).

[28] Mannaa, A. H., Zaky, R. R., Gomaa, E. A. & El-Hady, M. N. A. Estimation of the cyclic voltammetry parameters for pyridine-2,6-dicarbohydrazide and its interaction with CuCl_2_ in various solutions. *Monatshefte fur Chemie* **153**, 577–587. 10.1007/s00706-022-02947-3 (2022).

[29] Al-Harazie, A. G., Gomaa, E. A., Zaky, R. R. & Abd El-Hady, M. N. Cyclic voltammetry studies of malonamide hydrazone derivative and its electrochemical effect on CdCl_2_. *Electrochim. Acta* **476**, 143690. 10.1016/j.electacta.2023.143690 (2024).

[30] Albqmi, M., Elkanzi, N. A. A., Ali, A. M. & Abdou, A. Design, Characterization, and DFT exploration of new mononuclear Fe(III) and Co(II) complexes based on Isatin-hydrazone derivative: Anti-inflammatory profiling and molecular docking insights. *J. Mol. Struct.* **1319**, 139494. 10.1016/j.molstruc.2024.139494 (2025).

[31] Olasz, M., Peintler, G. & Schuszter, G. Determination of reaction stoichiometry by applying job’s method and digital image processing for precipitation reactions. *J. Chem. Educ.* **101**, 1280–1285. 10.1021/acs.jchemed.3c01306 (2024).

[32] Gomaa, A. I., Gomaa, E. A., Zaky, R. R. & Abd El-Hady, M. N. Synthesis of a pincer bis-hydrazone chelator for coordination, thermal, electrochemical, and biological investigations with CuII, CoII, and HgII. *J. Mol. Liq.* **408**, 10.1016/j.molliq.2024.125375 (2024).

[33] Krishna Priya, M., Revathi, B. K., Renuka, V., Sathya, S. & Samuel Asirvatham, P. Molecular structure, spectroscopic (FT-IR, FT-Raman,13C and 1H NMR) analysis, HOMO-LUMO energies, mulliken, MEP and thermal properties of new chalcone derivative by DFT calculation. *Mater. Today Proc.* **8**, 37–46. 10.1016/j.matpr.2019.02.078 (2019).

[34] Gökce, H., Bahçeli, S. & Alpaslan, G. Structural, vibrational, electronic, NLO and molecular docking analyses of two novel Cu(II) and Pd(II) complexes with pyridine and oxadiazole coordination. *Inorg. Chem. Commun.* **168**, 10.1016/j.inoche.2024.112852 (2024).

[35] Nageeb, A. S., Morsi, M. A., Gomaa, E. A., Hammouda, M. M. & Zaky, R. R. Comparison on biological inspection, optimization, cyclic voltammetry, and molecular docking evaluation of novel bivalent transition metal chelates of Schiff Base pincer ligand. *J. Mol. Struct.* **1300**, 137281. 10.1016/j.molstruc.2023.137281 (2024).

[36] El-Kot, D. A., Gomaa, E. A., El-askalany, A. M. H., Zaky, R. R. & Abd El-Hady, M. N. Design of a novel -NOON- tetradentate Schiff-base scaffold supported by α-tetralone and benzothiazole moieties with its Cu^2+^, Co^2+^, and Cd^2+^ chelates. *J. Mol. Struct.* **1278**, 134901. 10.1016/j.molstruc.2023.134901 (2023).

[37] Boukoucha, N. H. *et al.* Biological evaluation of a novel Schiff base ligand as an antioxidant agent: Synthesis, characterization and DFT computations of its Ni (II) and Cu (II) complexes. *J. Mol. Struct.* **1319**, 139505. 10.1016/j.molstruc.2024.139505 (2025).

[38] Pahonu, E. *et al.* Evaluation of antimicrobial, antitumor, antioxidant activities, and molecular docking studies of some Co (II), Cu (II), Mn (II), Ni (II), Pd (II), and Pt (II) complexes with a Schiff base derived from 2-chloro-5-(trifluoromethyl) aniline. *Appl. Organomet. Chem.* **39**, e7829. 10.1002/aoc.7829 (2025).

[39] Moreno-Narváez, M. E. *et al.* Glycoconjugate Pd (II) and Cu (II) complexes of fluorinated N, O Schiff base ligands for targeted cancer therapy: Synthesis, Characterization and In vitro Cytotoxic Activity Evaluation. *New J. Chem.* **49**, 5187–5199. 10.1039/D4NJ05181E (2025).

[40] Manjunath, M. *et al.* Sustainable synthesis of benzimidazole-based Schiff base using reusable CaAl2O4 nanophosphors catalyst: Insights into metal (II) complexes and DNA interactions. *Nucleosides, Nucleotides \& Nucleic Acids* 1–23. 10.1080/15257770.2025.2451375 (2025). 10.1080/15257770.2025.2451375 39827474

[41] Hosny, N. M., Samir, G. & Abdel-Rhman, M. H. N′-(Furan-2-ylmethylene)-2-hydroxybenzohydrazide and its metal complexes: Synthesis, spectroscopic investigations, DFT calculations and cytotoxicity profiling. *BMC Chem.* **18**, 1–17. 10.1186/s13065-023-01098-8 (2024). 38281963 10.1186/s13065-023-01098-8 PMC10823611

[42] Wu, Y. shu *et al.* Synthesis, crystal structure, DNA binding, and anticancer activity of the cobalt(II), nickel(II), and copper(II) complexes of 9-benzothiazolanthrahydrazone. *J. Mol. Struct.* **1299**, 137099. 10.1016/j.molstruc.2023.137099 (2024).

[43] Alorini, T., Daoud, I., Al-Hakimi, A. N. & Alminderej, F. Synthesis, characterization, anticancer activity, and molecular docking study of some metal complexes with a new Schiff base ligand. *J. Mol. Struct.* **1276**, 134785. 10.1016/j.molstruc.2022.134785 (2023). 10.1080/07391102.2023.2191725 36961125

[44] Göktürk, T. *et al.* Synthesis, structural investigations, DNA/BSA interactions, molecular docking studies, and anticancer activity of a new 1,4-disubstituted 1,2,3-triazole derivative. *ACS Omega* **8**, 31839–31856. 10.1021/acsomega.3c03355 (2023). 37692230 10.1021/acsomega.3c03355 PMC10483525

[45] Gomaa, A. I., Gomaa, E. A., Zaky, R. R. & Abd El-Hady, M. N. Design and synthesis of pyridine bis-hydrazone metal complexes of Co(II), Cu(II), and Hg(II): Spectral, Gaussian, electrochemical, biological, drug-likeness and molecular docking investigations. *Inorg. Chem. Commun.* **162**, 112188. 10.1016/j.inoche.2024.112188 (2024).

[46] El-Metwaly, N. *et al.* Synthesis and elucidation for new nanosized Cr(III)-pyrazolin complexes; crystal surface properties, antitumor simulation studies beside practical apoptotic path. *J. Inorg. Organomet. Polym. Mater.* **30**, 4142–4154. 10.1007/s10904-020-01561-2 (2020).

[47] Khedr, A. K., Zaky, R. R., Gomaa, E. A. & Abd El-Hady, M. N. Elucidation for coordination features of N-(benzothiazol-2-yl)-3-oxo-3-(2-(3-phenylallylidene)hydrazineyl)propanamide on Co^2+^, Ni^2+^and Cu^2+^: Structural description, DFT geometry optimization, cyclic voltammetry and biological inspection. *J. Mol. Liq.* **368**, 120613. 10.1016/j.molliq.2022.120613 (2022).

[48] Verquin, G. *et al.* EPR study of copper(II) complexes of hydroxysalen derivatives in order to be used in the DNA cleavage. *J. Photochem. Photobiol. B Biol.* **86**, 272–278. 10.1016/j.jphotobiol.2006.12.003 (2007). 10.1016/j.jphotobiol.2006.12.003 17227713

[49] Patel, S. K. *et al.* Mono- and binuclear copper(II) complexes with different structural motifs and geometries: Synthesis, spectral characterization, DFT calculations and superoxide dismutase enzymatic activity. *Polyhedron* **222**. 10.1016/j.poly.2022.115913 (2022).

[50] Hathaway, B. J. & Billing, D. E. The electronic properties and stereochemistry of mono-nuclear complexes of the copper(II) ion. *Coord. Chem. Rev.* **5**, 143–207. 10.1016/S0010-8545(00)80135-6 (1970).

[51] Fetoh, A., Mohammed, M. A., Youssef, M. M. & Abu El-Reash, G. M. Characterization, cyclic voltammetry and biological studies of divalent Co, Ni and Cu complexes of water-soluble, bioactive and photoactive thiosemicarbazone salt. *J. Mol. Liq.* **287**, 110958. 10.1016/j.molliq.2019.110958 (2019).

[52] Shenjie, L. *et al.* High-pressure steam treatment with Pt/TiO_2_ enhances the low temperature formaldehyde oxidation performance. *Appl. Surf. Sci.* **620**, 10.1016/j.apsusc.2023.156815 (2023).

[53] Tian, Q.-Q., Zhao, Z.-G. & Shi, Z.-C. A novel carbonothioate-based benzothiazole fluorescent probe for trace detection of mercury (II) in real water samples. *Inorganica Chim. Acta* **521**, 120349. 10.1016/j.ica.2021.120349 (2021).

[54] AbouElleef, E. M., Gomaa, E. A., Salem, M. A., Soud, M. R. & El-Ghobashy, M. A. Cyclic voltammetry analysis of mercuric chloride redox reactions with orange G dye. *J. Mol. Liq.* **414**, 126171. 10.1016/j.molliq.2024.126171 (2024).

[55] Rezaee, R., Montazer, M., Mianehro, A. & Mahmoudirad, M. Single-step synthesis and characterization of Zr-MOF onto wool fabric: Preparation of antibacterial wound dressing with high absorption capacity. *Fibers Polym.* **23**, 404–412. 10.1007/s12221-021-0211-y (2022).

[56] Abd El-Lateef, H. M., Khalaf, M. M., Kandeel, M. & Abdou, A. Synthesis, characterization, DFT, biological and molecular docking of mixed ligand complexes of Ni(II), Co(II), and Cu(II) based on ciprofloxacin and 2-(1H-benzimidazol-2-yl)phenol. *Inorg. Chem. Commun.* **155**, 111087. 10.1016/j.inoche.2023.111087 (2023).

[57] Tamer, Ö., Avcı, D., Dege, N. & Atalay, Y. Synthesis, crystal structure, photophysical properties, density functional theory calculations and molecular docking studies on Cd(II) complex of 4,4′-dimethyl-2,2′-dipyridyl. *J. Mol. Struct.* **1202**, 10.1016/j.molstruc.2019.127288 (2020).

[58] Soliman, A. Q. S., Abdel-Latif, S. A., Abdel-Khalik, S., Abbas, S. M. & Ahmed, O. M. Design, synthesis, structural characterization, molecular docking, antibacterial, anticancer activities, and density functional theory calculations of novel Mn^II^, Co^II^, Ni^II^, and Cu^II^ complexes based on pyrazolone-sulfadiazine azo-dye ligand. *J. Mol. Struct.* **1318**, 139402. 10.1016/j.molstruc.2024.139402 (2024).

[59] Adam, F. A., El-Reash, Y. G. A., Ghoniem, M. G. & Zaky, R. R. Investigate the effect of SiO_2_ on the chelation behavior of benzothiazole derivatives towards, Ni(П), Co(П), and Cu(П) metal ions: Quantitative structure, cyclic voltammetry and biological activity relationships. *SILICON* **16**, 2415–2439. 10.1007/s12633-023-02843-3 (2024).

[60] Mannaa, A. H., Zaky, R. R., Gomaa, E. A. & Abd El-Hady, M. N. Bivalent transition metal complexes of pyridine-2,6-dicarbohydrazide: Structural characterization, cyclic voltammetry and biological studies. *J. Mol. Struct.* **1269**, 133852. 10.1016/j.molstruc.2022.133852 (2022).

[61] Al-Harazie, A. G., Gomaa, E. A., Zaky, R. R. & Abd El-Hady, M. N. Spectroscopic characterization, cyclic voltammetry, biological investigations, Moe, and Gaussian calculations of VO(II), Cu(II), and Cd(II) Heteroleptic Complexes. *ACS Omega* **8**, 13605–13625. 10.1021/acsomega.2c07592 (2023). 10.1021/acsomega.2c07592 PMC10116629 37091434

[62] Wahba, R. H. *et al.* Electrochemical corrosion performance of N80 steel in acidized 10% HCl medium using 4-methyl-1-Phenyl-3-(p-tolyldiazenyl) −2,3-dihydro-1H-pyrrol-2-ol. *Heliyon* **11**, e42317. 10.1016/j.heliyon.2025.e42317 (2025). 39981360 10.1016/j.heliyon.2025.e42317 PMC11840194

[63] Akbari, Z. *et al.* Biological evaluation, DFT, MEP, HOMO-LUMO analysis and ensemble docking studies of Zn(II) complexes of bidentate and tetradentate Schiff base ligands as antileukemia agents. *J. Mol. Struct.* **1301**, 137400. 10.1016/j.molstruc.2023.137400 (2024).

[64] Arulmurugan, S. *et al.* Synthesis, solvent role in TD-DFT (IEFPCM model), fluorescence and reactivity properties, topology and molecular docking studies on sulfathiazole derivative. *J. Mol. Liq.* **400**, 124570. 10.1016/j.molliq.2024.124570 (2024).

[65] El-wahaab, B. A., Saad, M. Z., El-Desoky, S. I., Alrabie, A. & El-Shwiniy, W. H. Synthesis and characterization of new 4-amino-5-methyl-4H-1,2,4-triazole-3-thiol (MMTP) Schiff base for spectrophotometric detection of iron(III) and copper(II) ions in laboratory and different water samples: A biological and molecular docking exploration. *J. Mol. Struct.* **1318**, 139255. 10.1016/j.molstruc.2024.139255 (2024).

[66] Dinku, D. *et al.* Antimicrobial activities and docking studies of new Schiff base ligand and its Cu(II), Zn(II) and Ni (II) complexes: Synthesis and characterization. *Inorg. Chem. Commun.* **160**, 111903. 10.1016/j.inoche.2023.111903 (2024).

[67] Alcolea Palafox, M., Belskaya, N. P., Todorov, L. T. & Kostova, I. P. Structural study of a La(III) Complex of a 1,2,3-triazole ligand with antioxidant activity. *Antioxidants* **12**, 10.3390/antiox12101872 (2023). 10.3390/antiox12101872 PMC10604163 37891952

[68] Shi, F. *et al.* Ni/Mn-Complex-Tethered Tetranuclear polyoxovanadates: crystal structure and inhibitory activity on human hepatocellular carcinoma (HepG-2). *Molecules* **28**, 10.3390/molecules28196843 (2023). 10.3390/molecules28196843 PMC10574323 37836686

[69] Dhanya, T. M. *et al.* Unveiling the multifaceted bioactivity of copper (ii)–Schiff base complexes: A comprehensive study of antioxidant, anti-bacterial, anti-inflammatory, enzyme inhibition and cytotoxic potentials with DFT insights. *Dalt. Trans.* **54**, 3216–3234. 10.1039/D4DT02486A (2025). 10.1039/d4dt02486a 39820950

[70] Dhanya, T. M. *et al.* Synthesis, characterization, and biophysical and chemical properties of benzo [b] thiophene derivatives and their metal complexes. *New J. Chem.* **49**, 2850–2869. 10.1039/D4NJ04717F (2025).

[71] Shaaban, S. *et al.* Novel Fe (III), Cu (II), and Zn (II) chelates of organoselenium-based schiff base: design, synthesis, characterization, DFT, anticancer, antimicrobial, and antioxidant investigations. *Appl. Organomet. Chem.* **39**, e7776. 10.1002/aoc.7776 (2025).

[72] Abd El-Hady, M. N., Gomaa, E. A., Zaky, R. & Ela, S. E. Structural characterization, DFT geometry optimization, cyclic voltammetry and biological assay of (Tellurite-pyridine) Mixed Ligandcomplexesof Cd(II). *Research Squarre*. 10.21203/rs.3.rs-1203068/v1 (2022).

[73] Deswal, Y. *et al.* Metal complexes of 1,2,4-triazole based ligand: synthesis, structural elucidation, DFT calculations, alpha-amylase and alpha-glucosidase inhibitory activity along with molecular docking studies. *J. Inorg. Organomet. Polym. Mater.* **34**, 144–160. 10.1007/s10904-023-02808-4 (2024).

[74] Elgushe, S. M., El-Sonbati, A. Z., Diab, M. A., Gomaa, E. A. & AbouElleef, E. M. Eugenol’s electrochemical behavior, complexation interaction with copper chloride, antioxidant activity, and potential drug molecular docking application for Covid-19. *Colloids Surf. B Biointerfaces* **244**, 114194. 10.1016/j.colsurfb.2024.114194 (2024). 39226846 10.1016/j.colsurfb.2024.114194

[75] Adam, M. S. S., Elsawy, H., Sedky, A., Makhlouf, M. M. & Taha, A. Catalytic potential of sustainable dinuclear (Cu^2+^ and ZrO^2+^) metal organic incorporated frameworks with comprehensive biological studies. *J. Taiwan Inst. Chem. Eng.* **144**, 104747. 10.1016/j.jtice.2023.104747 (2023).

[76] Zaky, R. & Fekri, A. Solid state ball milling as a green approach to prepare Cu(II) complexes: Structural, spectral, DFT, and DNA studies. *New J. Chem.* **41**, 4555–4563. 10.1039/c7nj00840f (2017).

[77] Alshamrani, M. Selected metal (Au, Ag, and Cu) complexes of n-heterocyclic ligands as potential anticancer agents: A review. *Anticancer. Agents Med. Chem.* **25**, 11. 10.2174/0118715206331002241119145651 (2025). 10.2174/0118715206331002241119145651 39773056

[78] BURAN, K. Metal complexes of sulfamethazine/benzoin-based Schiff base ligand: synthesis, characterization, DFT calculations, and antimicrobial activities. *Turkish J. Biol.* **49**, 118–126. 10.55730/1300-0152.2729 (2025). 10.55730/1300-0152.2729 PMC11913352 40104579

[79] Khan, T. *et al.* Synthesis, computational drug likeness, DFT studies and in vitro biological activity evaluation of some heteronuclear complexes of functionalized (E)-2-(butan-2-ylidene) hydrazinecarbothioamide ligand. *Vietnam J. Chem.* **61**, 514–531. 10.1002/vjch.202200233 (2023).

[80] Belkhir-Talbi, D. *et al.* Synthesis, characterization, theoretical studies, ADMET and drug-likeness analysis: Electrochemical and biological activities of metal complexes of 3-(2-hydroxybenzoyl)-2H-chromen-2-one. *J. Mol. Struct.* **1179**, 495–505. 10.1016/j.molstruc.2018.11.035 (2019).

