# Supplementary material for: Bivalent transition metal complexes of triazole pyridine Schiff base with theoretical and biological investigations
Source: Sci Rep. 2025 Aug 25;15:31192. doi: 10.1038/s41598-025-15782-3 (PMC12379659; doi:10.1038/s41598-025-15782-3)
Supplement: Supplementary file 1 — Supplementary Information. [file 41598_2025_15782_MOESM1_ESM.docx]

**Bivalent Transition Metal Complexes of Triazole Pyridine Schiff base with Theoretical and Biological Investigations**

**Abdullah H. Mannaa^1^*, Esam A. Gomaa^1^, Rania R. Zaky^1^, Eslam A. Ghaith^1^, Mahmoud N. Abd El-Hady^1^**

**^1^** Chemistry Department, Faculty of Science, Mansoura University, Mansoura, Egypt

***Corresponding Author:** E-mail; [Abdullahmannaa@std.mans.edu.eg](mailto:Abdullahmannaa@std.mans.edu.eg)

| - **IR** | FTIR spectrophotometer, Model: Bruker (Invenio S, Germany), Spectral Range: 400 – 4000 cm-1, Spectral Resolution: 4 cm-1. | **** |
| --- | --- | --- |
| - **UV-Vis** | UV visible Spectrophotometer  (190 nm – 1100 nm), edinburgh Instruments DS5 Dual Beam | **** |
| - **NMR** | EM-390 (200 MHz) on  a Varian Mercury-300  instrument (Switzerland)  to recorded ^1^H NMR &  ^13^C NMR spectra in DMSO | 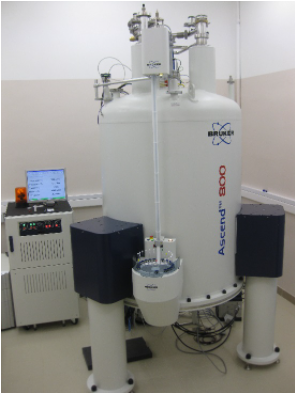 |
| - **Elemental analyses** | Elemental analyses were carried out using FLASH 2000 CHNS/O analyzer, Thermo Scientific at the Regional Center for Mycology and Biotechnology (RCMB). |  |
| - **EPR** | BRUKER EMX EPR spectroscopy located in the National center for Radiation Research and technology (NCRRT) | **** |
| - **XRD** | XRD D8 Advanced BRUKER Co. (Germany), wavelength 1.54A, 40kv, 40mA used to detect the X-ray diffraction patterns | 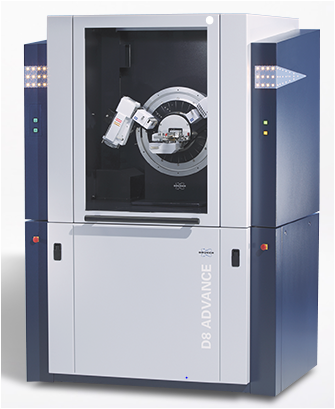 |
| - **TGA** | TGA-50 thermogravimetric  analyzer (shimadzu). | **** |
| - **MS** | Mass spectrum was carried out using EI mode on Direct Inlet part to mass analyzer in Thermo Scientific GCMS model ISQ (RCMB), Al-Azhar University, Cairo |  |
| - **DY2000 potentiostat** | DY2000 potentiostat(USA) |  |
| - **Magnetism** | Magnetic susceptibility balance (Johnson Matthey Wayne, Pennsylvania, USA) used to detect the magnetic properties | 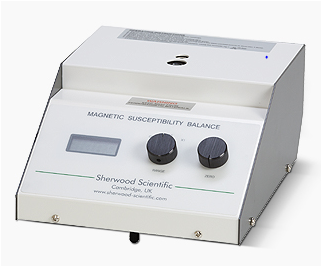 |
| **Table S1.** The Equipment's used in the structural characterization | | |

**Scheme S1.** Antimicrobial activity screening assay

**Scheme S2.** Antioxidant activity screening assay *via* ABTS method

**Scheme S3.** Colorimetric DNA-binding assay for investigated compounds

**Scheme S4**. The initial screening and cell viability by MTT assay

| **Compound** | **ν(C=N)_azo_** | **ν(C=N)_triazine_** | **ν(NH_2_)** | **ν(SH)** | **ν(ring breathing)_py_** | **ν(COO^-^)_sym_** | **ν(COO^-^)_asy_** | **ν(M-N)** |
| --- | --- | --- | --- | --- | --- | --- | --- | --- |
| H_2_TAP | 1607 | 1641 | 3302, 3279 | 2890 | 859 | -- | -- | -- |
| [Cu(H_2_TAP)(H_2_O)(Cl_2_)].H_2_O | 1595 | 1640 | 3242, 3219 | 2894 | 866 | -- | -- | 498 |
| [Mn(H_2_TAP)(H_2_O)(Cl_2_)].H_2_O | 1600 | 1637 | 3246, 3184 | 2883 | 867 | -- | -- | 481 |
| [Hg(H_2_TAP) (OAc) _2_)].2H_2_O | 1610 | 1636 | 3287, 3271 | 2859 | 860 | 1530 | 1453 | 477 |

**Table S2.** Most important IR bonds of H_2_P ligand and its complexes

|  |
| --- |
|  |
|  |
|  |
| **Fig. S1.** IR of )**a**) H_2_TAP ligand, (**b**) [Cu(H_2_TAP)(H_2_O)(Cl_2_)].H_2_O, (**c**) [Mn(H_2_TAP)(H_2_O)(Cl_2_)].H_2_O, and (**d**) [Hg(H_2_TAP)_2_(Cl_2_)].2H_2_O complexes |

|  |  |
| --- | --- |
| **a** | **b** |
|  |  |
| **c** | **d** |

**Fig. S2.** The Experimental and theoretical IR spectrum of )**a**) H_2_TAP ligand, (**b**) [Cu(H_2_TAP)(H_2_O)(Cl_2_)].H_2_O, (**c**) [Mn(H_2_TAP)( H_2_O)(Cl_2_)].H_2_O and (**d**) [Hg(H_2_TAP)(OAc)_2_].2H_2_O.

| **** |
| --- |
| **Fig. S3**. Mass spectrum of H_2_TAP ligand |

| ****  **(a)** |
| --- |
| ****  **(b)** |
| **Fig. S4**. ^1^H NMR of )**a**) H_2_TAP ligand and (**b**) [(Hg)(H_2_TAP)_2_(Cl_2_)].2H_2_O complex |

|  |
| --- |
| (**a**) |
|  |
| (**b**) |
| **Fig. S5**. ^13^C NMR of )**a**) H_2_TAP ligand and (**b**) [Hg(H_2_TAP)_2_(Cl_2_)].2H_2_O complex |

| **Compound** | **H^1^ NMR** | | | | | **C^13^ NMR** | | | | |
| --- | --- | --- | --- | --- | --- | --- | --- | --- | --- | --- |
|  | δ(N**H_2_**) | δ(N**H**) _triazine_ | δ(S**H**) | **δ(**C**H_3_)** | δ(C_6_**H_4_**) | δ(**C**=N) _azomethine_ | δ(**C**=N) _triazine_ | δ(S-**C**=N) | **δ(C**H_3_**)** | δ(**C_6_**H_4_) |
| H_2_TAP | 4.037 | 9.909 | 14.369 | 2.520-2.383 | 7.55-8.73 | 138.27 | 149.75 | 165.12 | 12.39 | 149.55, 146.15, 128.28, 125.27, 122.61 |
| [Hg(H_2_TAP)(OAc)_2_].2H_2_O | 4.022 | 10.343 | 14.998 | 2.399-2.490 | 7.425-8.611 | 138.76 | 153.99 | 147.43 | 12.37 | 150.82, 148.42, 137.15, 124.37, 120.79 |

**Table S3.** H^1^ and C^13^ NMR chemical shifts of H_2_TAP ligand and Hg-complex (ppm)

| **Compound** | **Wavenumber  (cm^-1^)** | **magnetic moment (**B.M**)** |
| --- | --- | --- |
| H_2_TAP ligand | -- | -- |
| [Cu(H_2_TAP)(H_2_O)(Cl_2_)].H_2_O | 16129, 14184 | 1.91 |
| [Mn(H_2_TAP)(H_2_O)(Cl_2_)].H_2_O | 27777, 19084 | 5.82 |

**Table S4**. Electronic spectra and magnetic moment of Cu^II^, and Mn^II^ complexes

| ****  **a** | ****  **b** |
| --- | --- |
| ****  **c** | ****  **d** |
| **Fig. S6.** UV of )**a**) H_2_TAP ligand, (**b**) [Cu(H_2_TAP)(H_2_O)(Cl_2_)].H_2_O, (**c**) [Mn(H_2_TAP)(H_2_O)(Cl_2_)].H_2_O, and (**d**) [Hg(H_2_TAP)_2_(Cl_2_)].2H_2_O complexes | |

| Compound | g*_\|\|_* | g_⊥_ | A*_\|\|_***(cm^-1^)** | A_⊥_**(cm^-1^)** | G | F | *α* ^2^ | *β* ^2^ |
| --- | --- | --- | --- | --- | --- | --- | --- | --- |
| [Cu(H_2_TAP)(H_2_O)(Cl_2_)].H_2_O | 2.142 | 2.064 | 0.010 | 0.093 | 2.284 | 205.96 | 0.495 | 0.937 |

**Table S5**. ESR Parameters for Cu^II^ Complex

**Table S6.** Thermal behavior of metal complexes of H_2_TAP

| **Compound**  **(Molecular weight)** | **Temp. Range, (^°^C)** | **stage** | **Decomposition product(s) loss** | **Formula weight** | **Weight %**  **Found (Calcd)** |
| --- | --- | --- | --- | --- | --- |
| [Cu(H_2_TAP)(H_2_O)(Cl_2_)].H_2_O  (419.77) | 27-195 | 1^st^ | Removal of 2H_2_O | 36.03 | 8.58 (8.58) |
|  | 195-255 | 2^nd^ | Removal of Cl_2_ | 70.91 | 16.93 (16.89) |
|  | 255-328 | 3^rd^ | Removal of C_2_H_3_N_4_S | 115.13 | 27.42 (27.43) |
|  | 328-799 | 4^th^ | Removal of C_7_H_4_N | 102.12 | 24.32 (24.33) |
|  | >799 | Residue | Leaving Cu(NH_2_)_2_ | 95.59 | 22.75 (22.77) |
| [Mn(H_2_TAP)(Cl_2_)(H_2_O)].H_2_O  (411.16) | 27-175 | 1^st^ | Removal of H_2_O | 18.01 | 4.22 (4.38) |
|  | 175-338 | 2^nd^ | Removal of H_2_O + Cl_2_ | 88.92 | 21.99 (21.63) |
|  | 338-550 | 3^rd^ | Removal of C_2_H_3_N_4_S | 115.13 | 28.11 (28.00) |
|  | 550-799 | 4^th^ | Removal of C_7_H_4_N | 102.12 | 24.30 (24.84) |
|  | >799 | Residue | Leaving Mn(NH_2_)_2_ | 86.94 | 21.38 (21.15) |

| **a** |  |
| --- | --- |
| **b** |  |
|  | **Fig. S7**. TGA of (**a**) [Cu(H_2_TAP)(H_2_O)(Cl_2_)].H_2_O, and (**b**) [Mn(H_2_TAP)(H_2_O)(Cl_2_)].H_2_O complexes |

|   **(a)** |
| --- |
|   **(b)** |

**Scheme S5.** The outline decomposition pattern of TG analysis for (**a**) [Cu(H_2_TAP)( H_2_O)(Cl_2_)].H_2_O and (**b**) [Mn(H_2_TAP)(H_2_O) (Cl_2_)].H_2_O

| **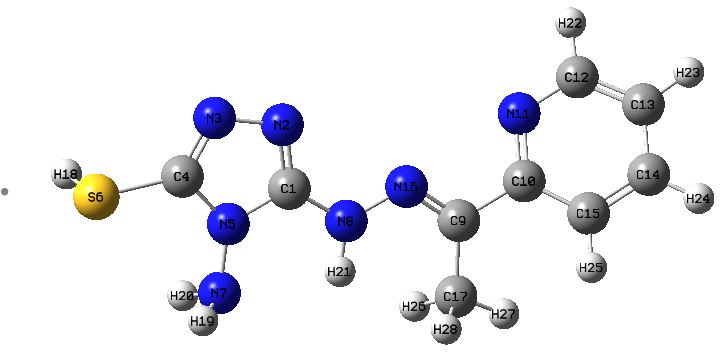** | **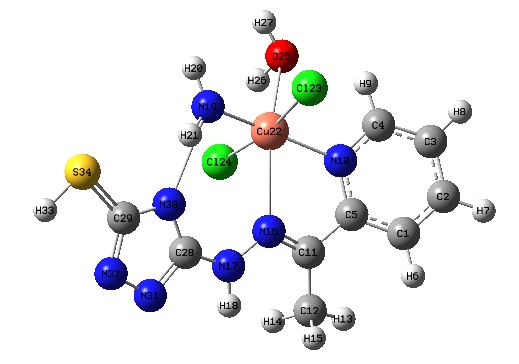** |
| --- | --- |
| **a** | **b** |
| **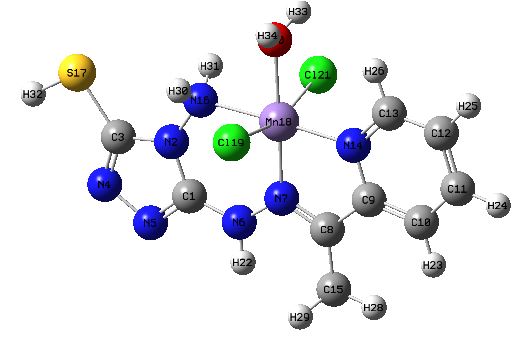** | **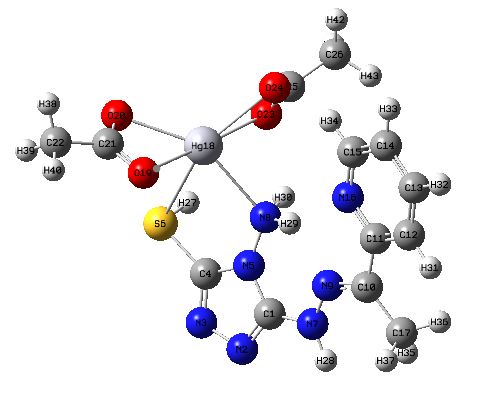** |
| **c** | **d** |
| **Fig. S8**. Modelling of )**a**) H_2_TAP ligand, (**b**) [Cu(H_2_TAP)(H_2_O)(Cl_2_)].H_2_O, (**c**) [Mn(H_2_TAP)(H_2_O)(Cl_2_)].H_2_O and (**d**) [Hg(H_2_TAP)(OAc)_2_].2H_2_O complexes | |

| **No.** | **Ligand H_2_TAP** | | **Cu- H_2_TAP complex** | | **Mn- H_2_TAP complex** | | **Hg- H_2_TAP complex** | |
| --- | --- | --- | --- | --- | --- | --- | --- | --- |
|  | **Atom** | **Charge** | **atom** | **Charge** | **atom** | **Charge** | **atom** | **Charge** |
| 1 | C | 0.10381 | C | -0.18503 | C | 0.14133 | C | 0.60496 |
| 2 | N | -0.36048 | C | -0.19865 | N | -0.02332 | N | -0.17995 |
| 3 | N | 0.01347 | H | 0.23958 | C | -0.17989 | N | -0.01592 |
| 4 | C | -0.06797 | H | 0.21064 | N | -0.02554 | C | -0.24015 |
| 5 | N | 0.00599 | N | -0.13022 | N | -0.07544 | N | -0.45047 |
| 6 | S | 0.14469 | C | 0.34210 | N | -0.28665 | S | 0.25605 |
| 7 | N | -0.66778 | C | -0.66889 | N | -0.13328 | N | -0.47842 |
| 8 | N | -0.23174 | H | 0.22084 | C | 0.23588 | N | -0.67572 |
| 9 | C | -0.2575 | N | -0.1832 | C | 0.34546 | N | -0.11757 |
| 10 | C | 0.19705 | N | -0.33707 | C | -0.33922 | C | 0.27192 |
| 11 | N | -0.06989 | H | 0.31174 | C | -0.19785 | C | 0.18047 |
| 12 | C | -0.19858 | N | -0.37083 | C | -0.20593 | C | -0.31189 |
| 13 | C | 0.14695 | H | 0.15183 | C | -0.17191 | C | -0.05909 |
| 14 | C | -0.36486 | H | 0.22994 | N | -0.15131 | C | -0.18816 |
| 15 | C | 0.14062 | Cu | -0.10613 | C | -0.76359 | C | -0.02065 |
| 16 | N | 0.07981 | Cl | -0.21168 | N | -0.58564 | N | -0.28666 |
| 17 | C | -0.53857 | Cl | -0.28269 | S | 0.06040 | C | -0.67635 |
| 18 | H | 0.10125 | O | -0.60547 | Mn | -0.17207 | Hg | 0.81412 |
| 19 | H | 0.28443 | H | 0.41624 | Cl | -0.26070 | O | -0.40023 |
| 20 | H | 0.31616 | H | 0.37456 | O | -0.61694 | O | -0.49747 |
| 21 | H | 0.23388 | S | 0.03855 | Cl | -0.27527 | C | 0.43119 |
| 22 | H | 0.14178 | C | -0.07402 | H | 0.32139 | C | -0.60061 |
| 23 | H | 0.13149 | N | -0.09973 | H | 0.23102 | O | -0.45255 |
| 24 | H | 0.13558 | N | -0.17350 | H | 0.23816 | O | -0.62444 |
| 25 | H | 0.11577 | N | -0.16185 | H | 0.22908 | C | 0.55865 |

**Table S7**. Selected Mulliken Charges of H_2_TAP and its complexes

| **No.** | **Ligand H_2_TAP** | | **Cu- H_2_TAP complex** | | **Mn- H_2_TAP complex** | | **Hg- H_2_TAP complex** | |
| --- | --- | --- | --- | --- | --- | --- | --- | --- |
|  | **Bond name** | **Bond length(A°)** | **Bond name** | **Bond length(A°)** | **Bond name** | **Bond length(A°)** | **Bond name** | **Bond length(A°)** |
| 1 | C(17)-H(28) | 1.099 | O(17)-H(31) | 1.031 | O(20)-H(34) | 1.047 | S(6)-H(44) | 1.366 |
| 2 | C(17)-H(27) | 1.09 | O(17)-H(30) | 1.025 | O(20)-H(33) | 1.048 | C(26)-H(43) | 1.111 |
| 3 | C(17)-H(26) | 1.099 | Cu(16)-Cl(21) | 2.187 | Mn(18)-Cl(21) | 2.17 | C(22)-C(21) | 1.541 |
| 4 | C(15)-H(25) | 1.085 | Cu(16)-Cl(20) | 2.187 | O(20)-Mn(18) | 1.86 | O(20)-C(21) | 1.501 |
| 5 | C(14)-H(24) | 1.086 | N(18)-Cu(16) | 1.909 | Cl(19)-Mn(18) | 2.174 | O(19)-C(21) | 1.305 |
| 6 | C(14)-C(15) | 1.394 | O(17)-Cu(16) | 1.859 | S(17)-H(32) | 1.344 | O(24)-Hg(18) | 2.161 |
| 7 | C(13)-H(23) | 1.085 | C(15)-H(29) | 1.114 | N(16)-H(31) | 1.094 | O(23)-Hg(18) | 2.119 |
| 8 | C(13)-C(14) | 1.394 | C(15)-H(28) | 1.113 | N(16)-H(30) | 1.077 | O(20)-Hg(18) | 2.16 |
| 9 | C(12)-H(22) | 1.088 | C(15)-H(27) | 1.114 | N(16)-Mn(18) | 1.904 | O(19)-Hg(18) | 2.091 |
| 10 | C(12)-C(13) | 1.399 | N(14)-Cu(16) | 1.391 | C(15)-H(29) | 1.113 | C(17)-H(37) | 1.112 |
| 11 | N(11)-C(12) | 1.335 | C(13)-H(26) | 1.108 | C(15)-H(28) | 1.113 | C(17)-H(36) | 1.123 |
| 12 | C(10)-C(15) | 1.407 | C(13)-N(14) | 1.279 | C(15)-H(27) | 1.114 | C(17)-H(35) | 1.103 |
| 13 | C(10)-N(11) | 1.346 | C(12)-H(25) | 1.103 | N(14)-Mn(18) | 1.859 | N(8)-H(29) | 1.098 |
| 14 | C(9)-C(17) | 1.513 | C(12)-C(13) | 1.346 | C(9)-C(10) | 1.346 | N(8)-Hg(18) | 2.215 |
| 15 | C(9)-N(16) | 1.292 | C(11)-H(24) | 1.102 | C(8)-C(15) | 1.514 | N(7)-H(28) | 1.044 |
| 16 | C(9)-C(10) | 1.49 | C(8)-C(15) | 1.509 | C(8)-C(9) | 1.349 | N(7)-N(9) | 1.299 |
| 17 | N(8)-H(21) | 1.016 | C(8)-C(9) | 1.334 | N(7)-Mn(18) | 1.864 | S(6)-H(27) | 1.374 |
| 18 | N(8)-N(16) | 1.353 | N(7)-Cu(16) | 1.399 | N(7)-C(8) | 1.276 | S(6)-Hg(18) | 2.519 |
| 19 | N(7)-H(20) | 1.018 | N(7)-C(8) | 1.274 | N(6)-H(22) | 1.05 | N(5)-N(8) | 1.427 |
| 20 | N(7)-H(19) | 1.018 | N(6)-H(22) | 1.052 | N(6)-N(7) | 1.242 | C(4)-S(6) | 1.481 |
| 21 | S(6)-H(18) | 1.353 | N(6)-N(7) | 1.25 | N(4)-N(5) | 1.255 | C(4)-N(5) | 1.269 |
| 22 | N(5)-N(7) | 1.398 | N(4)-N(5) | 1.257 | C(3)-S(17) | 1.81 | N(3)-C(4) | 1.253 |
| 23 | C(4)-S(6) | 1.764 | C(3)-S(19) | 1.809 | C(3)-N(4) | 1.264 | N(2)-N(3) | 1.261 |
| 24 | C(4)-N(5) | 1.396 | C(3)-N(4) | 1.266 | N(2)-N(16) | 1.4 | C(1)-N(7) | 1.361 |
| 25 | N(3)-C(4) | 1.306 | N(2)-N(18) | 1.386 | N(2)-C(3) | 1.269 | N(5)-C(1) | 1.27 |

**Table S8.** Selected bond Lengths of H_2_TAP and its complexes

| **No.** | **H_2_TIS Ligand** | | **Cu-H_2_TAP complex** | | **Mn-H_2_TAP complex** | | **Hg-H_2_TAP complex** | |
| --- | --- | --- | --- | --- | --- | --- | --- | --- |
|  | **Bond angle name** | **Bond angle(°)** | **Bond angle name** | **Bond angle(°)** | **Bond angle name** | **Bond angle(°)** | **Bond angle name** | **Bond angle(°)** |
| 1 | H(28)-C(17)-H(27) | 107.995 | H(34)-S(19)-C(3) | 109.097 | H(34)-O(20)-H(33) | 158.93 | H(43)-C(26)-H(42) | 116.315 |
| 2 | H(28)-C(17)-H(26) | 107.498 | H(33)-N(18)-H(32) | 73.603 | H(34)-O(20)-Mn(18) | 100.869 | C(26)-C(25)-O(24) | 123.59 |
| 3 | H(28)-C(17)-C(9) | 111.983 | H(33)-N(18)-Cu(16) | 91.979 | H(33)-O(20)-Mn(18) | 100.197 | C(26)-C(25)-O(23) | 134.158 |
| 4 | H(27)-C(17)-H(26) | 106.758 | H(33)-N(18)-N(2) | 95.388 | Cl(21)-Mn(18)-O(20) | 83.976 | O(24)-C(25)-O(23) | 100.273 |
| 5 | H(27)-C(17)-C(9) | 111.816 | H(32)-N(18)-Cu(16) | 128.584 | Cl(21)-Mn(18)-Cl(19) | 167.814 | C(25)-O(24)-Hg(18) | 93.76 |
| 6 | H(26)-C(17)-C(9) | 110.548 | H(32)-N(18)-N(2) | 108.444 | Cl(21)-Mn(18)-N(16) | 93.139 | C(25)-O(23)-Hg(18) | 107.182 |
| 7 | C(9)-N(16)-N(8) | 117.803 | Cu(16)-N(18)-N(2) | 122.114 | Cl(21)-Mn(18)-N(14) | 96.357 | O(24)-Hg(18)-S(6) | 156.04 |
| 8 | H(25)-C(15)-C(14) | 120.003 | H(31)-O(17)-H(30) | 170.584 | Cl(21)-Mn(18)-N(7) | 97.015 | O(23)-Hg(18)-O(20) | 62.428 |
| 9 | H(25)-C(15)-C(10) | 120.767 | H(31)-O(17)-Cu(16) | 93.802 | O(20)-Mn(18)-Cl(19) | 83.872 | O(23)-Hg(18)-O(19) | 105.959 |
| 10 | C(14)-C(15)-C(10) | 119.209 | Cl(20)-Cu(16)-O(17) | 167.276 | O(20)-Mn(18)-N(16) | 84.146 | O(23)-Hg(18)-N(8) | 62.354 |
| 11 | H(24)-C(14)-C(15) | 120.29 | Cl(20)-Cu(16)-N(14) | 92.654 | O(20)-Mn(18)-N(14) | 106.194 | O(23)-Hg(18)-S(6) | 108.216 |
| 12 | H(24)-C(14)-C(13) | 120.847 | Cl(20)-Cu(16)-N(7) | 95.464 | O(20)-Mn(18)-N(7) | 171.988 | O(20)-Hg(18)-O(19) | 46.429 |
| 13 | C(15)-C(14)-C(13) | 118.861 | N(18)-Cu(16)-O(17) | 89.767 | Cl(19)-Mn(18)-N(16) | 84.728 | O(20)-Hg(18)-N(8) | 120.69 |
| 14 | H(23)-C(13)-C(14) | 121.595 | N(18)-Cu(16)-N(14) | 172.378 | Cl(19)-Mn(18)-N(14) | 87.899 | O(20)-Hg(18)-S(6) | 133.525 |
| 15 | H(23)-C(13)-C(12) | 120.551 | N(18)-Cu(16)-N(7) | 87.414 | H(30)-N(16)-N(2) | 84.842 | O(19)-Hg(18)-N(8) | 167.099 |
| 16 | C(14)-C(13)-C(12) | 117.854 | O(17)-Cu(16)-N(14) | 92.641 | Mn(18)-N(16)-N(2) | 120.854 | O(19)-Hg(18)-S(6) | 106.233 |
| 17 | H(22)-C(12)-C(13) | 120.327 | O(17)-Cu(16)-N(7) | 95.053 | H(29)-C(15)-H(28) | 105.104 | N(8)-Hg(18)-S(6) | 83.648 |
| 18 | H(22)-C(12)-N(11) | 115.719 | N(14)-Cu(16)-N(7) | 99.567 | H(29)-C(15)-H(27) | 108.57 | H(28)-N(7)-C(1) | 106.149 |
| 19 | C(13)-C(12)-N(11) | 123.952 | H(29)-C(15)-H(28) | 108.726 | H(29)-C(15)-C(8) | 112.881 | N(9)-N(7)-C(1) | 148.025 |
| 20 | C(12)-N(11)-C(10) | 118.267 | C(15)-C(8)-C(9) | 126.74 | H(28)-C(15)-H(27) | 108.707 | H(44)-S(6)-H(27) | 35.225 |
| 21 | C(15)-C(10)-N(11) | 121.853 | C(15)-C(8)-N(7) | 124.636 | H(28)-C(15)-C(8) | 110.542 | H(44)-S(6)-Hg(18) | 83.695 |
| 22 | C(15)-C(10)-C(9) | 120.667 | C(9)-C(8)-N(7) | 108.06 | H(27)-C(15)-C(8) | 110.809 | H(44)-S(6)-C(4) | 83.532 |
| 23 | N(11)-C(10)-C(9) | 117.476 | Cu(16)-N(7)-C(8) | 109.427 | Mn(18)-N(14)-C(13) | 120.277 | H(27)-S(6)-Hg(18) | 108.037 |
| 24 | C(17)-C(9)-N(16) | 123.509 | Cu(16)-N(7)-N(6) | 119.109 | Mn(18)-N(14)-C(9) | 106.841 | H(27)-S(6)-C(4) | 111.831 |
| 25 | C(17)-C(9)-C(10) | 120.172 | C(8)-N(7)-N(6) | 103.411 | C(13)-N(14)-C(9) | 120.023 | Hg(18)-S(6)-C(4) | 81.196 |

**Table S9.** Selected bond Angles of H_2_TAP and its complexes

| **a** |  |
| --- | --- |
| **b** |  |
| **c** |  |
| **d** |  |

**Fig. S9.** Selected bond Lengths of )**a**) H_2_TAP ligand, (**b**) [Cu(H_2_TAP)(H_2_O)(Cl_2_)].H_2_O, (**c**) [Mn(H_2_TAP)(H_2_O)(Cl_2_)].H_2_O and (**d**) [Hg(H_2_TAP)(OAc)_2_].2H_2_O complexes

| **a** |  |
| --- | --- |
| **b** |  |
| **c** |  |
| **d** |  |

**Fig. S10**. Selected bond Angles of )**a**) H_2_TAP ligand, (**b**) [Cu(H_2_TAP)(H_2_O)(Cl_2_)].H_2_O, (**c**) [Mn(H_2_TAP)(H_2_O)(Cl_2_)].H_2_O and (**d**) [Hg(H_2_TAP)(OAc)_2_].2H_2_O complexes

| **Compound** | **Gram-negative bacteria** | | | **Gram-positive bacteria** | | | | **fungus** | | |
| --- | --- | --- | --- | --- | --- | --- | --- | --- | --- | --- |
|  | ***E. coli*** | |  |  | ***S. aureus*** | | | ***C. albicans*** | |  |
|  | **inhibition zone (mm)** | **% Activity index** | **MIC** | **inhibition zone**  **(mm)** | | **% Activity index** | **MIC** | **inhibition zone**  **(mm)** | **% Activity index** | **MIC** |
| H_2_TAP | 11 | 42.3 | 8 | 9 | | 37.5 | 4 | 10 | 37.0 | 8 |
| [Cu(H_2_TAP)(H_2_O)(Cl_2_)].H_2_O | 13 | 50.0 | 4 | 12 | | 50.0 | 2 | 15 | 55.5 | 4 |
| [Mn(H_2_TAP)(H_2_O)(Cl_2_)].H_2_O | 4 | 15.4 | 16 | 5 | | 20.8 | 8 | 12 | 44.4 | 4 |
| [Hg(H_2_TAP)(OAc)_2_].2H_2_O | 8 | 30.8 | 16 | 7 | | 29.2 | 16 | 7 | 25.9 | 32 |
| **Ciprofloxacin** | 26 | 100 | 0.5 | 24 | | 100 | 0.5 | -- | -- | -- |
| **Colitrimazole** | -- | -- | -- | -- | | -- | -- | 27 | 100 | 1 |

**Table S10**. Anti-microbial assay of H_2_TAP ligand and its complexes

| **Compound** | **Concentration (µM)** | | | | | | |
| --- | --- | --- | --- | --- | --- | --- | --- |
|  | 10 | 20 | 40 | 60 | 80 | 100 | **IC_50_** |
|  | **% Inhibition** | | | | | |  |
| Vitamin C | 24.2 | 37.4 | 54.9 | 66.7 | 78.5 | 94.3 | 29.47±0.17 |
| H_2_TAP | 18.0 | 29.3 | 40.5 | 55.0 | 64.8 | 81.7 | 45.10±0.25 |
| [Cu(H_2_TAP)(H_2_O)(Cl_2_)].H_2_O | 17.5 | 23.4 | 34.3 | 37.2 | 44.9 | 63.2 | 82.21±0.49 |
| [Mn(H_2_TAP)(H_2_O)(Cl_2_)].H_2_O | 20.8 | 34.7 | 49.1 | 58.6 | 67.7 | 84.1 | 37.23±0.20 |
| [Hg(H_2_TAP)(OAc)_2_].2H_2_O | 14.4 | 25.1 | 32.0 | 46.7 | 58.1 | 72.3 | 59.31±0.31 |

**Table S11.** All the results of Inhibition effect (I %) and IC_50_ for the antioxidant ABTS method

| **Compound** | **Concentration (*µ*M)** | | | | | | | **In vitro Cytotoxicity IC_50_ (µM)*** |
| --- | --- | --- | --- | --- | --- | --- | --- | --- |
|  | 1.5625 | 3.125 | 6.25 | 12.5 | 25 | 50 | 100 |  |
|  | **Average of Relative viability of cells (%)** | | | | | | |  |
| **Cisplatin** | 98.2 | 81.4 | 63.7 | 42.9 | 35.0 | 24.1 | 19.3 | **12.89±1.0** |
| **Doxorubicin** | 71.2 | 57.6 | 45.8 | 28.3 | 14.1 | 11.2 | 6.3 | **4.50±0.2** |
| **Sorafenib** | 95.4 | 72.9 | 61.3 | 37.2 | 25.7 | 16.6 | 9.2 | **9.18±0.6** |
| H_2_TAP | 100 | 95.6 | 82.3 | 67.4 | 49.8 | 35.8 | 26.8 | **28.56±1.9** |
| [Cu(H_2_TAP)(H_2_O)(Cl_2_)].H_2_O | 100 | 100 | 95.6 | 81.3 | 68.1 | 55.6 | 42.8 | **65.40±3.6** |
| [Mn(H_2_TAP)(H_2_O)(Cl_2_)].H_2_O | 100 | 84.8 | 69.3 | 41.4 | 34.1 | 27.3 | 19.2 | **13.88±1.2** |
| [Hg(H_2_TAP)(OAc)_2_].2H_2_O | 100 | 100 | 96.8 | 74.9 | 60.8 | 49.5 | 34.1 | **46.86±2.7** |

***IC50 (µM)**: 1 – 10 (very strong). 11 – 20 (strong). 21 – 50 (moderate). 51 – 100 (weak) and above 100 (non-cytotoxic)

**Table S12.** All the results of Cell viability % and IC_50_ for the HePG-2 cell line

| **DNA-active compound** | **DNA/methyl green (IC_50_, µM)** |
| --- | --- |
| **DOX** | 31.54±1.5 |
| **H_2_TAP** | 45.31±2.2 |
| **[Cu(H_2_TAP)(H_2_O)(Cl_2_)].H_2_O** | 73.24±3.1 |
| **[Mn(H_2_TAP)(H_2_O)(Cl_2_)].H_2_O** | 39.65±1.9 |
| **[Hg(H_2_TAP)(OAc)_2_].2H2O** | 61.80±2.7 |

**Table S13**. DNA/methyl green colourimetric assay of the DNA-binding compounds

| **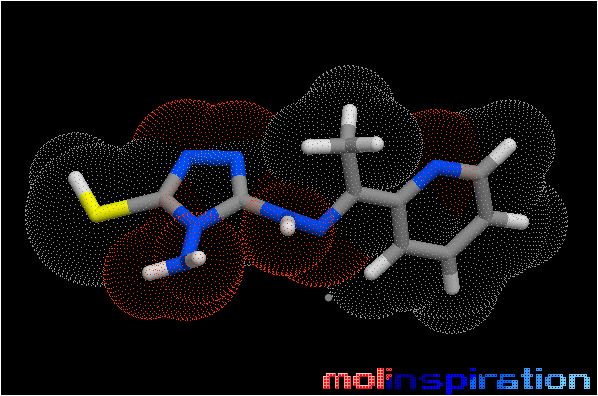** | **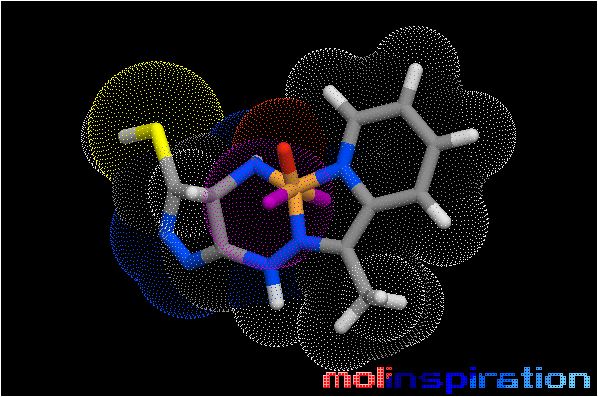** |
| --- | --- |
| H_2_TAP ligand | [Cu(H_2_TAP)(H_2_O)(Cl_2_)].H_2_O |
| **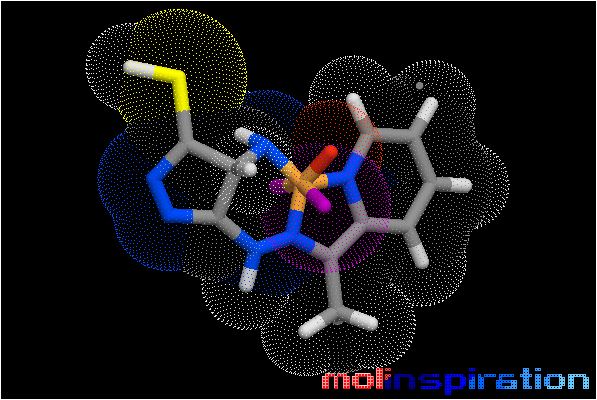** | **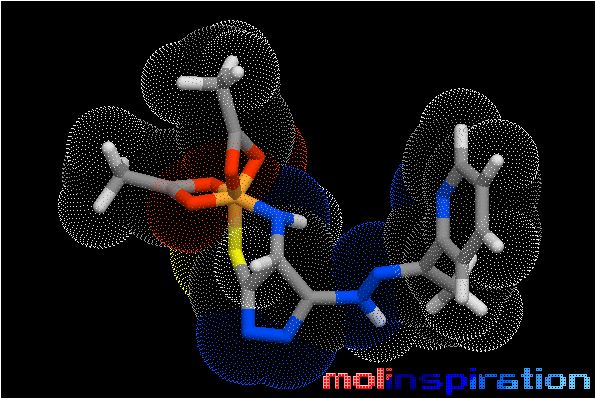** |
| [Mn(H_2_TAP)(H_2_O)(Cl_2_)].H_2_O | [Hg(H_2_TAP)(OAc)_2_(Cl_2_)].2H_2_O |
| **Fig. S11.** 3D Structures resulting from Molinspiration software of H_2_TAP ligand and its complexes. | |
